# Identification of a TGF-β/SMAD/lnc-UTGF positive feedback loop and its role in hepatoma metastasis

**DOI:** 10.1038/s41392-021-00781-3

**Published:** 2021-11-17

**Authors:** Meng-Zhi Wu, Yi-chuan Yuan, Bi-Yu Huang, Jin-Xi Chen, Bin-Kui Li, Jian-Hong Fang, Shi-Mei Zhuang

**Affiliations:** 1 MOE Key Laboratory of Gene Function and Regulation, School of Life Sciences, Collaborative Innovation Center for Cancer Medicine, Sun Yat-sen University, Xin Gang Xi Road 135#, Guangzhou, 510275 P. R. China; 2 Department of Hepatobiliary Surgery, Sun Yat-sen University Cancer Center, Dong Feng Road East 651#, Guangzhou, 510060 P. R. China

**Keywords:** Metastasis, Non-coding RNAs

## Abstract

Aberrant activation of the TGF-β/SMAD signaling pathway is often observed in hepatocellular carcinoma (HCC). Whether lncRNA regulates the TGF-β/SMAD signaling remains largely unknown. Here, we identified an oncogenic lncRNA that was upregulated in HCC and was transcriptionally induced by TGF-β (named lnc-UTGF, lncRNA upregulated by TGF-β). Upon TGF-β stimulation, SMAD2/3 bound to the lnc-UTGF promoter and activated lnc-UTGF expression. In turn, the TGF-β/SMAD signaling was augmented by overexpressing lnc-UTGF, but was inhibited by silencing lnc-UTGF. Mechanism investigations revealed that lnc-UTGF interacted with the mRNAs of SMAD2 and SMAD4 via complementary base-pairing, resulting in enhanced stability of SMAD2/4 mRNAs. These data suggest a novel TGF-β/SMAD/lnc-UTGF positive feedback circuitry. Subsequent gain- and loss-of-function analyses disclosed that lnc-UTGF promoted the migration and invasion of hepatoma cells, and this effect of lnc-UTGF was attenuated by repressing SMAD2/4 expression or by mutating the SMAD2/4-binding sites in lnc-UTGF. Studies using mouse models further confirmed that in vivo metastasis of hepatoma xenografts was inhibited by silencing lnc-UTGF, but was enhanced by ectopic expression of lnc-UTGF. The lnc-UTGF level was positively correlated with the SMAD2/4 levels in xenografts. Consistently, we detected an association of lnc-UTGF upregulation with increase of SMAD2, SMAD4, and their metastasis effector SNAIL1 in human HCC. And high lnc-UTGF level was also significantly associated with enhanced metastasis potential, advanced TNM stages, and worse recurrence-free survival. *Conclusion*: there exists a lnc-UTGF-mediated positive feedback loop of the TGF-β signaling and its deregulation promotes hepatoma metastasis. These findings may provide a new therapeutic target for HCC metastasis.

## Introduction

Transforming growth factor-β (TGF-β) is a multifunctional cytokine that plays an essential role in cell proliferation and differentiation, and in morphogenesis, tissue homeostasis and regeneration.^1,2^ In the canonical TGF-β signaling pathway, TGF-β binds to TGFβRII to trigger the phosphorylation of TGFβRI, which results in phosphorylation and activation of SMAD2 and SMAD3. The activated-SMAD2/3 cooperates with SMAD4 to form a SMAD transcription complex, which translocates into nucleus and activates the transcription of its downstream genes.^3,4^ The homeostasis of the TGF-β/SMAD signaling is regulated by feedback loops, like TGF-β/SMAD7 negative feedback loop, in which SMAD7 is transactivated by SMAD2/3 and then represses the TGF-β/SMAD signaling by binding to TGFβRI/II.^5^ It has been shown that disruption of the TGF-β signaling contributes to tumorigenesis, fibrotic disorders, immune malfunctions, etc.^2,6,7^ The TGF-β signaling pathway is frequently activated in different cancer types and facilitates tumor metastasis by inducing the transcription of pro-metastasis genes and promoting the migration and invasion of cancer cells.^2,8^ Given the important role of the TGF-β signaling in tumor development, identifying novel feedback loops that modulate TGF-β/SMAD signaling and affect tumor development may provide targets for cancer therapy.

Hepatocellular carcinoma (HCC) is a worldwide common malignancy with high mortality. Early intrahepatic recurrence/metastasis is a frequent event and represents the major cause of the dismal outcome of HCC patients, whereas extrahepatic metastasis occurs much less frequently and is not the leading cause of HCC death.^9–11^ HCC is mainly developed from chronic hepatitis. A high level of TGF-β, as a consequence of chronic liver damage and the activation of fibroblast cells, is detected in HCC tissues and is correlated with poor prognosis of patients.^10,12–14^ Long non-coding RNAs (lncRNAs) are newly discovered non-protein-coding transcripts longer than 200 nucleotides. LncRNAs may interact with DNA, RNA or proteins to regulate various cell activities, like proliferation, apoptosis, and motility.^15,16^ Growing evidences indicate that dysfunction of lncRNAs plays vital roles in different physiological and pathological processes, including tumor development.^17–19^ To date, whether lncRNA regulates the TGF-β/SMAD signaling in HCC is still unknown.

In this study, we disclose a novel positive feedback loop of the TGF-β/SMAD pathway, that is, the TGF-β/SMAD signaling induces the transcription of lnc-UTGF (representing for lncRNA upregulated by TGF-β), whereas lnc-UTGF in turn promotes the TGF-β/SMAD signaling by stabilizing the mRNAs of SMAD2 and SMAD4. We further show that upregulation of lnc-UTGF in hepatoma cells results in abnormal activation of this positive feedback loop and thus enhanced HCC metastasis.

## Results

### The transcription of lnc-UTGF is induced by the TGF-β/SMAD signaling

**Fig. 1:**
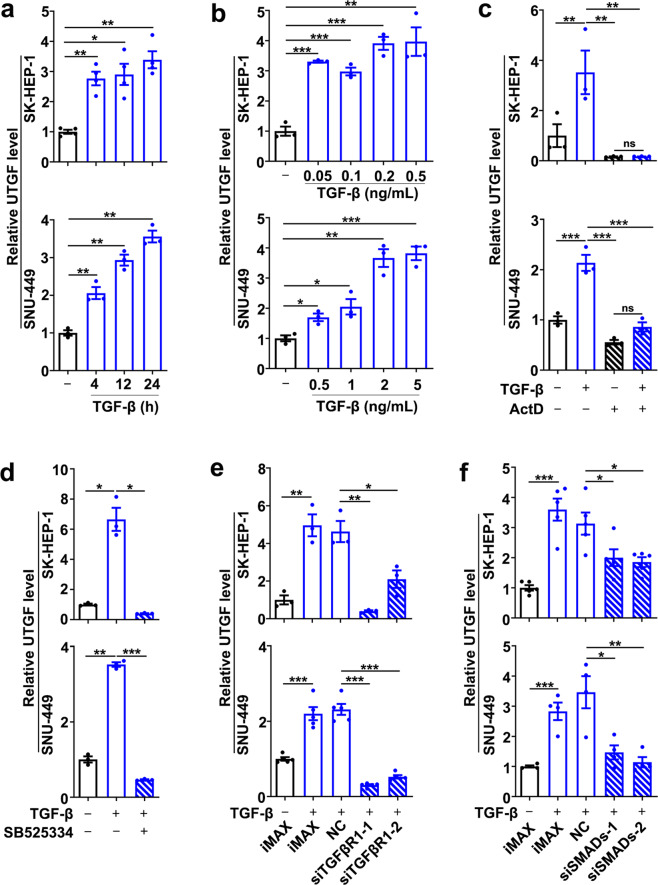
TGF-β activates lnc-UTGF transcription via the canonical TGF-β/SMAD signaling pathway. **a**, **b** TGF-β increased lnc-UTGF level in a time- and dose-dependent manner. SK-HEP-1 or SNU-449 cells were untreated (-) or treated with 2 ng/ml TGF-β for the indicated time (**a**) or with the indicated dose of TGF-β for 24 h (**b**). **c** Treatment with transcription inhibitor abolished the TGF-β-induced lnc-UTGF expression. Cells were incubated without or with TGF-β or actinomycin-D (ActD) for 6 h. **d**-**f** Inhibition of TGFβR1 or simultaneous knockdown of SMAD2/3/4 attenuated the TGF-β-induced lnc-UTGF expression. For **d**, cells were incubated without or with TGF-β or TGFβR1 inhibitor (SB525334) for 12 h. For **e**–**f**, cells transfected with the indicated RNA duplexes were incubated without or with TGF-β for 24 h. iMAX, cells treated with Lipofectamine RNAiMAX without RNA duplexes. NC, cells transfected with negative control RNA duplex. siTGFβR1-1 and siTGFβR1-2, cells transfected with siRNA targeting different sequences of TGFβR1. siSMADs-1 and siSMADs-2, cells transfected with the mixture of siRNAs targeting SMAD2, SMAD3, and SMAD4. Lnc-UTGF level was detected by qPCR analysis, and U6 was used as an internal control. + or −, cells with (+) or without (−) the indicated treatment. Error bars: SEM from at least three independent experiments. *, *P* < 0.05; **, *P* < 0.01; ***, *P* < 0.001; ns, not significant

To identify lncRNAs that are induced by TGF-β, bioinformatics analysis was conducted by using three transcriptome profiles from the cells with or without TGF-β treatment. As shown, two lncRNAs, AP000695.4 and LINC00312, displayed a more than 2-fold increase upon TGF-β exposure in all three data sets (Supplementary Fig. S1a). And only AP000695.4 (named lnc-UTGF for lncRNA upregulated by TGF-β) was significantly upregulated as early as 4 h after TGF-β treatment and at a time- and dose-dependent manner (Fig. 1a, b and Supplementary Fig. S1b), suggesting lnc-UTGF as an early response gene to TGF-β exposure. Further investigations revealed that the role of TGF-β in increasing lnc-UTGF level was abrogated by blocking gene transcription with actinomycin D (ActD, Fig. 1c). Furthermore, either inhibitor of TGFβR1 (SB525334) or siRNA targeting TGFβR1 (Supplementary Fig. S2a, b) or simultaneous knockdown of SMAD2/3/4 (Supplementary Fig. S2c, d) significantly attenuated the TGF-β-induced lnc-UTGF expression (Fig. 1d–f). These findings indicate that TGF-β may promote lnc-UTGF transcription via the canonical TGF-β/SMAD signaling pathway.

### SMAD2 and SMAD3 directly bind to and activate the lnc-UTGF promoter

**Fig. 2:**
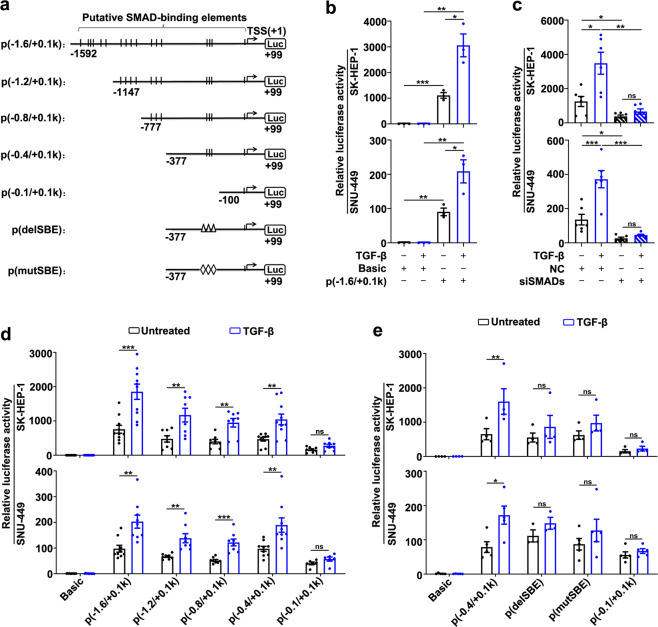
The lnc-UTGF promoter is activated by the TGF-β/SMAD signaling. **a** Schematic diagram of firefly luciferase reporters carrying the indicated DNA fragments of the lnc-UTGF promoter. Arrow designates the transcription direction of lnc-UTGF. Short vertical line: the putative SMAD-binding element (SBE). Triangle (∆): deletion of the SMAD-binding element (delSBE). Rhombus (◊): mutation of the SMAD-binding element (mutSBE). TSS, transcriptional start site, Luc, luciferase gene. **b** TGF-β enhanced the activity of the lnc-UTGF promoter. Cells that were transfected with the indicated vectors for 36 h were untreated or treated with TGF-β for another 12 h. **c** Simultaneous knockdown of SMAD2/3/4 abrogated the effect of TGF-β in enhancing the lnc-UTGF promoter activity. Cells were transfected with NC or siSMAD2/3/4 for 12 h, followed by co-transfection with p(−1.6/+0.1k) and pRL-CML vectors for 24 h, then untreated or treated with TGF-β for another 12 h. **d** Sequential 5′-end deletion analysis showed that the −0.4 to −0.1-kb region of the lnc-UTGF promoter contained the TGF-β response elements. **e** Deletion or mutation of putative SBEs in the lnc-UTGF promoter abrogated the response of p(−0.4/+0.1k) to TGF-β. For **d**, **e**, cells that were transfected with the indicated vectors for 36 h were untreated or treated with TGF-β for another 12 h. The promoter activity was examined by luciferase activity assay. Basic, pGL3-basic vector. Error bars: SEM from at least three independent experiments. *, *P* < 0.05; **, P < 0.01; ***, *P* < 0.001; ns, not significant

**Fig. 3:**
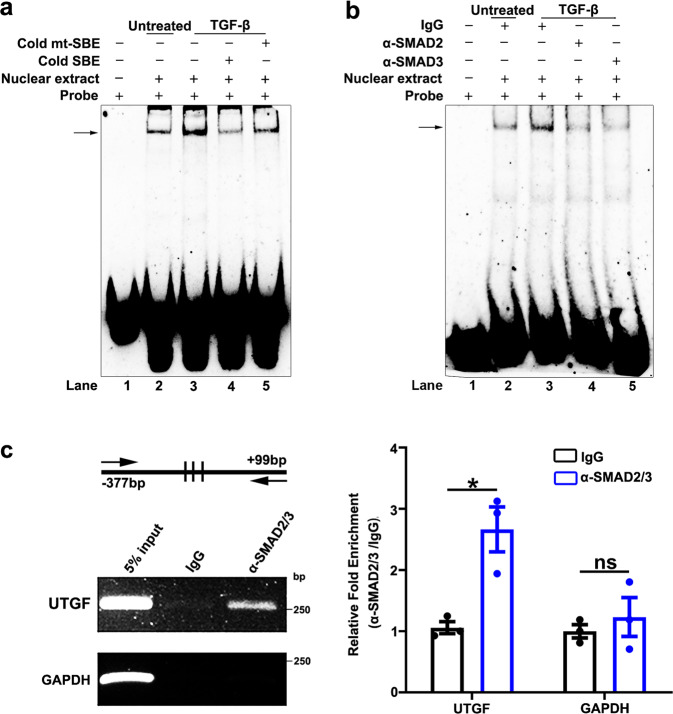
SMAD2 and SMAD3 directly bind to the lnc-UTGF promoter in vitro and in vivo. **a**, **b** EMSA and antibody-supershift assays verified the in vitro interaction of SMAD2/3 with SBEs in the lnc-UTGF promoter. DNA–protein complexes are indicated by the arrows. Nuclear extracts were isolated from SK-HEP-1 cells untreated or treated with TGF-β for 2 h. Probe: biotin-labeled oligonucleotides that comprise SBE sequence in the lnc-UTGF promoter. Cold SBE: unlabeled oligonucleotides that contain consensus SMAD-binding element (SBE). Cold mt-SBE: unlabeled oligonucleotides that contain mutant SBE. **c** SMAD2/3 interacted with the lnc-UTGF promoter in vivo. SK-HEP-1 cells were incubated with TGF-β for 1 h before ChIP assay. The antibody-precipitated DNAs were amplified by semi-quantitative PCR for 35 cycles (*left panel*) or by qPCR (*right panel*). Arrows: the PCR primers. 5% input: 5% of the total DNAs were amplified to serve as the control for DNA content. Values shown are fold enrichment of α-SMAD2/3-precipitated DNA relative to the isotype-matched IgG control. The promoter region of GAPDH was used as a negative control. + or − , cells with (+) or without (−) the indicated treatment. Error bars: SEM from three independent experiments. *, *P* < 0.05, ns, not significant

To explore how TGF-β induced lnc-UTGF transcription, we firstly identified the transcription start site (TSS, assigned as +1-bp, chr 21: 36430302) of lnc-UTGF and characterized lnc-UTGF as a 955-nt transcript that was conserved among human, chimp, and rhesus, but was not conserved between human and mouse or rat (Supplementary Fig. S3a-c). Further investigations revealed that lnc-UTGF was located in both cytoplasmic and nuclear compartments (Supplementary Fig. S4a) and had no protein-coding capacity (Supplementary Fig. S4b, c). We then explored whether lnc-UTGF was a direct transcriptional target of SMAD complex. Chromatin immunoprecipitation (ChIP)-sequencing data from ENCODE showed that the histone modifications associated with an active promoter, including H3K4Me1, H3K4Me3, and H3K27Ac, were enriched within the 1.6-kb genomic region upstream of the TSS of lnc-UTGF (Supplementary Fig. S5), and 17 SMAD-binding elements (SBEs) were predicted within this region (Fig. 2a). Experimentally, the promoter reporter p(−1.6/+0.1k) that contained the −1592 to +99-bp sequence of lnc-UTGF showed an obviously increased luciferase activity (Fig. 2b, bar 3 vs. bar 1). Moreover, TGF-β enhanced the p(−1.6/+0.1k) activity (Fig. 2b, bar 4 vs. bar 3), and this stimulatory effect was blocked when SMAD2/3/4 were simultaneously knocked down (Fig. 2c). Further sequential 5′-end deletion analysis showed that TGF-β increased the luciferase activity of the reporter containing the −1592 ~ −377-bp sequence, but had no effect on the reporter with the −100 ~ +99-bp region of lnc-UTGF (Fig. 2d), suggesting that the −377 ~ −100-bp of the lnc-UTGF promoter may contain TGF-β response elements. Indeed, deletion or mutation of all three putative SBEs within this region abrogated the response of p(−0.4/+0.1k) to TGF-β (Fig. 2a, e). EMSA revealed that the biotin-labeled probe carrying these three putative SBEs could form specific complexes with nuclear proteins, as manifested by the appearance of a specific band (Fig. 3a, *lane* 2), and the intensity of this band increased upon TGF-β stimulation (Fig. 3a, *lane* 3), whereas this promotive effect of TGF-β was blocked by an unlabeled oligonucleotide with two classic SBEs (Fig. 3a, *lane* 4) but remained unchanged in the presence of an unlabeled oligonucleotide with mutant SBE sequence (Fig. 3a, *lane* 5). Antibody supershift assay showed that pre-incubation with anti-SMAD2 or anti-SMAD3 antibody significantly reduced the band intensity of the probe–protein complexes (Fig. 3b). Furthermore, ChIP assays disclosed that the −377 ~ +99-bp fragment of lnc-UTGF promoter, but not the promoter of a negative control gene GAPDH, was enriched in the DNAs that were precipitated by anti-SMAD2/3 antibody (Fig. 3c). These results indicate a direct interaction between SMAD2/3 and the lnc-UTGF promoter in vitro and in vivo.

Taken together, upon stimulation of TGF-β, SMAD2/3 may directly bind to the lnc-UTGF promoter and induce lnc-UTGF transcription.

### Lnc-UTGF promotes the TGF-β/SMAD signaling by increasing SMAD2/SMAD4 levels

**Fig. 4:**
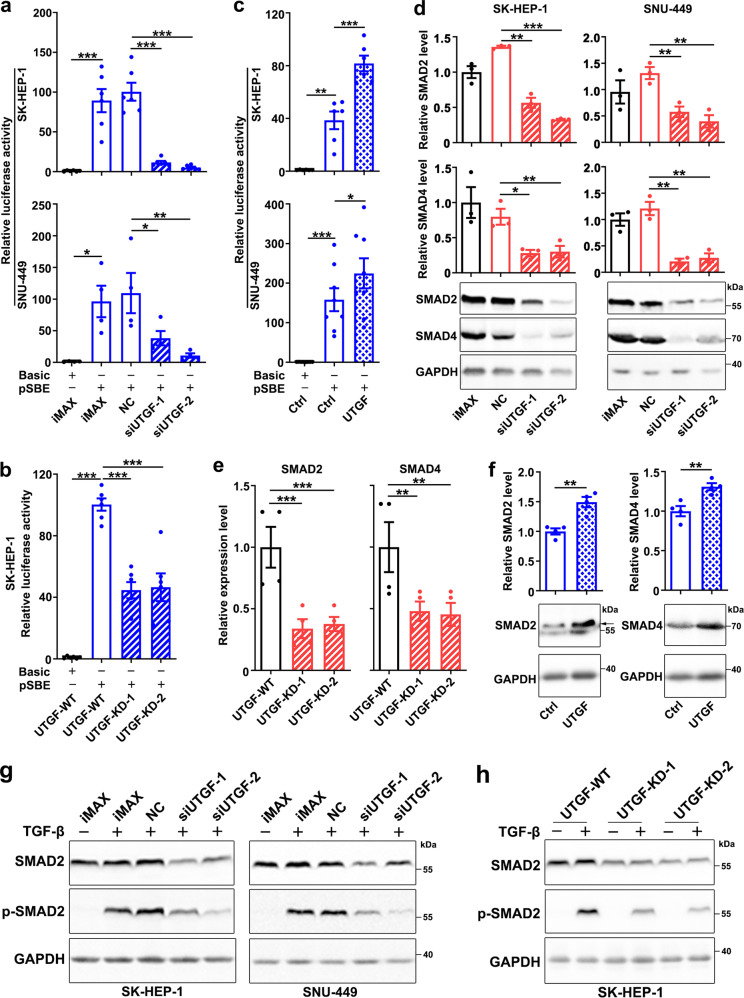
Lnc-UTGF promotes the TGF-β/SMAD signaling by increasing SMAD2 and SMAD4 levels. **a**, **b** Silencing lnc-UTGF abrogated the effect of TGF-β in stimulating pSBE reporter activity. Cells transfected with the indicated siRNA for 12 h (**a**), or two sublines with heterozygous knockout of lnc-UTGF (UTGF-KD-1 and -2) and their control line (UTGF-WT; **b**) were analyzed. **c** Overexpressing lnc-UTGF promoted the effect of TGF-β in stimulating pSBE reporter activity. Cells stably expressing lnc-UTGF and its control cells (Ctrl) were examined. For **a**–**c**, cells were transfected with the indicated plasmids for 36 h, then incubated with TGF-β for 12 h before luciferase activity analysis. Basic, pGL3-basic vector; pSBE, a luciferase reporter bearing twelve tandem SBEs. **d**, **e** Silencing lnc-UTGF decreased the mRNA and protein levels of SMAD2 and SMAD4. Cells transfected with the indicated RNA duplexes for 48 h (**d**), or sublines with heterozygous knockout of lnc-UTGF and their control line (**e**) were analyzed by qPCR and Western blotting. **f** Overexpressing lnc-UTGF increased the mRNA and protein levels of SMAD2 and SMAD4. Cells stably expressing lnc-UTGF and its control cells (Ctrl) were treated with 0.5 ng/ml TGF-β for 18 h, then subjected to qPCR and Western blotting. **g**, **h** Silencing lnc-UTGF decreased the levels of total and phosphorylated SMAD2. Cells transfected with the indicated RNA duplexes for 48 h (**g**), or sublines with heterozygous knockout of lnc-UTGF and their control cells (**h**), were untreated or treated with TGF-β1 for 1 h, followed by Western blotting. siUTGF-1 and siUTGF-2, cells transfected with siRNA targeting different sequences of lnc-UTGF. U6 and GAPDH were used as internal controls for qPCR and Western blotting, respectively. + or −, cells with (+) or without (−) the indicated treatment. Error bars: SEM from three independent experiments. *, *P* < 0.05; **, *P* < 0.01; ***, *P* < 0.001

To explore the function of lnc-UTGF in HCC, we performed Gene Set Enrichment Analysis (GSEA) to identify lnc-UTGF-associated pathways by using the transcriptome data of human HCC tissues derived from The Cancer Genome Atlas (TCGA). The results revealed that genes regulating the cellular response to TGF-β stimulus and genes in the TGF-β receptor pathways were significantly enriched in the group with high lnc-UTGF level compared with the group with low lnc-UTGF level (Supplementary Fig. 6). Therefore, we experimentally assessed whether lnc-UTGF had a feedback control on the TGF-β/SMAD signaling. The transactivation activity of TGF-β signaling was first measured by using pSBE, a luciferase reporter bearing SMAD-binding elements. As shown, TGF-β stimulated the pSBE activity and this effect was significantly attenuated in the cells transfected with siRNA targeting lnc-UTGF (siUTGF; Fig. 4a; Supplementary Fig. S7a) or in the cells with heterozygous knockout (Fig. 4b; Supplementary Fig. S7b) or stable knockdown of lnc-UTGF (Supplementary Fig. S7c, d). On the other hand, ectopic expression of lnc-UTGF (Supplementary Fig. S7e) enhanced the effect of TGF-β in stimulating pSBE activity (Fig. 4c). We then examined the levels of the key components in the canonical TGF-β/SMAD pathway, including TGFβR1, SMAD2, SMAD3, SMAD4, and SMAD7. Silencing lnc-UTGF significantly reduced both mRNA and protein levels of SMAD2 and SMAD4 (Fig. 4d, e, Supplementary Fig. S8a), but did not affect the levels of other molecules examined (Supplementary Fig. S8b, c). Consistently, overexpressing lnc-UTGF increased both mRNA and protein levels of SMAD2 and SMAD4 (Fig. 4f). Phosphorylation of SMAD2 and SMAD3, and the nuclear translocation of SMAD2/3/4 complex are the key events of TGF-β signaling activation. As shown, silencing lnc-UTGF reduced the levels of total and phosphorylated SMAD2 but not SMAD3 (Fig. 4g, h; Supplementary Fig. S9a, b). Moreover, both nuclear and cytoplasmic SMAD2 and SMAD4 proteins were decreased by silencing lnc-UTGF (Supplementary Fig. S9c). These results indicate that lnc-UTGF may increase the SMAD2/4 levels and exert a positive feedback regulation on the TGF-β/SMAD signaling.

### Lnc-UTGF interacts with the mRNAs of SMAD2 and SMAD4 and enhances their stability

**Fig. 5:**
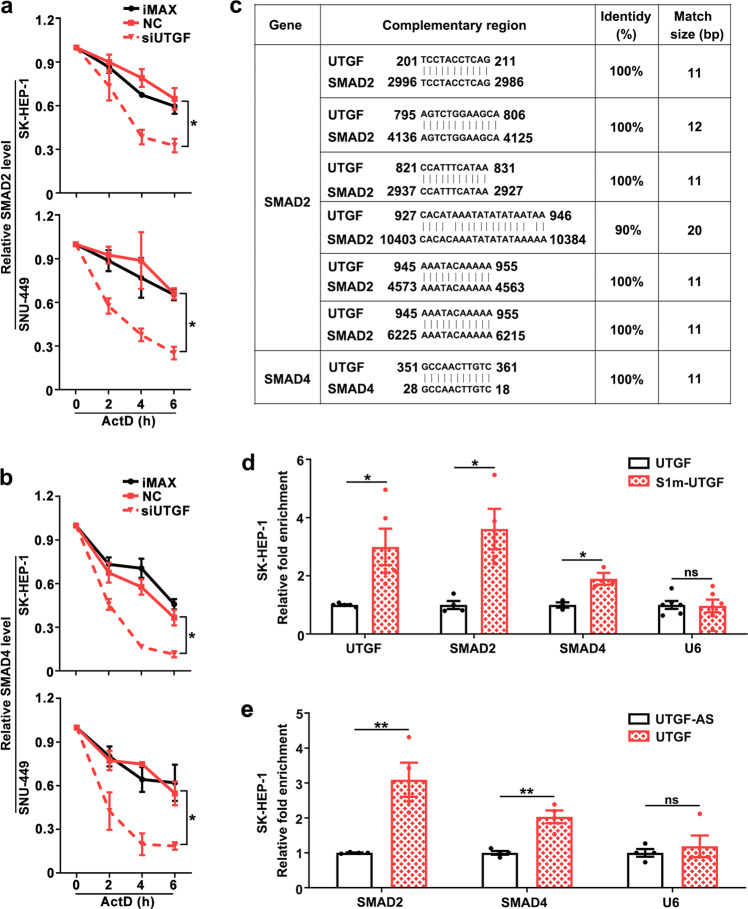
Lnc-UTGF interacts with the mRNAs of SMAD2 and SMAD4 and enhances their stability. **a**–**b** Silencing lnc-UTGF shortened the half-life of mature mRNAs of SMAD2 and SMAD4. Cells transfected with the indicated RNA duplexes were treated with actinomycin-D (ActD) for the indicated time, followed by qPCR analysis. U6 was used as an internal control. **c** Complementary base-pairing between lnc-UTGF and SMAD2/4 mRNAs. The putative complementary sequences were predicted by BLAST (https://blast.ncbi.nlm.nih.gov/). **d**–**e** Lnc-UTGF was physically associated with the mRNAs of SMAD2 and SMAD4. For **d**, the SK-UTGF and SK-S1m-UTGF sublines were applied to S1m-tagged RNA affinity purification assays. The indicated RNAs in the S1m-pull-down precipitates and in the input were detected by qPCR. The RNA level of the pull-down product was corrected by that in the input. The mean value of the adjusted RNA level in the pull-down product of the SK-UTGF group was set as relative RNA level 1. For **e**, the RNA pulled down by biotin-labeled lnc-UTGF or by biotin-labeled antisense RNA of lnc-UTGF (UTGF-AS, negative control) were examined by qPCR. U6, negative control. Error bars: SEM from three independent experiments. *, *P* < 0.05; **, *P* < 0.01; ns, not significant

We further explored how lnc-UTGF upregulated the expression of SMAD2 and SMAD4. As shown, silencing lnc-UTGF did not affect the precursor mRNA levels of SMAD2 and SMAD4 (Supplementary Fig. S10a, b), but shortened the half-life of mature mRNAs of SMAD2 and SMAD4 (Fig. 5a, b), indicating that lnc-UTGF may post-transcriptionally regulate the SMAD2 and SMAD4 levels. Bioinformatics analysis revealed six highly complementary regions between lnc-UTGF and SMAD2-mRNA, and one complementary region between lnc-UTGF and SMAD4-mRNA (Fig. 5c). To test whether there was a direct interaction between lnc-UTGF and SMAD2/4 mRNA, the full-length lnc-UTGF was tagged with a modified streptavidin-binding RNA aptamer S1m and then transfected into cells. Compared with the untagged-UTGF group, the mRNAs of SMAD2 and SMAD4 were significantly enriched in the S1m-UTGF precipitates (Fig. 5d), whereas the negative control U6 was not enriched in the S1m-UTGF precipitates, indicating a specific interaction between lnc-UTGF and SMAD2/4 mRNAs. These interactions were further validated by affinity pull-down of cellular mRNAs of SMAD2 and SMAD4 using in vitro transcribed lnc-UTGF (Fig. 5e). These data suggest that lnc-UTGF may stabilize the mRNAs of SMAD2/4 via a direct interaction of complementary base-pairing.

### Lnc-UTGF promotes tumor metastasis by enhancing the TGF-β/SMAD signaling

**Fig. 6:**
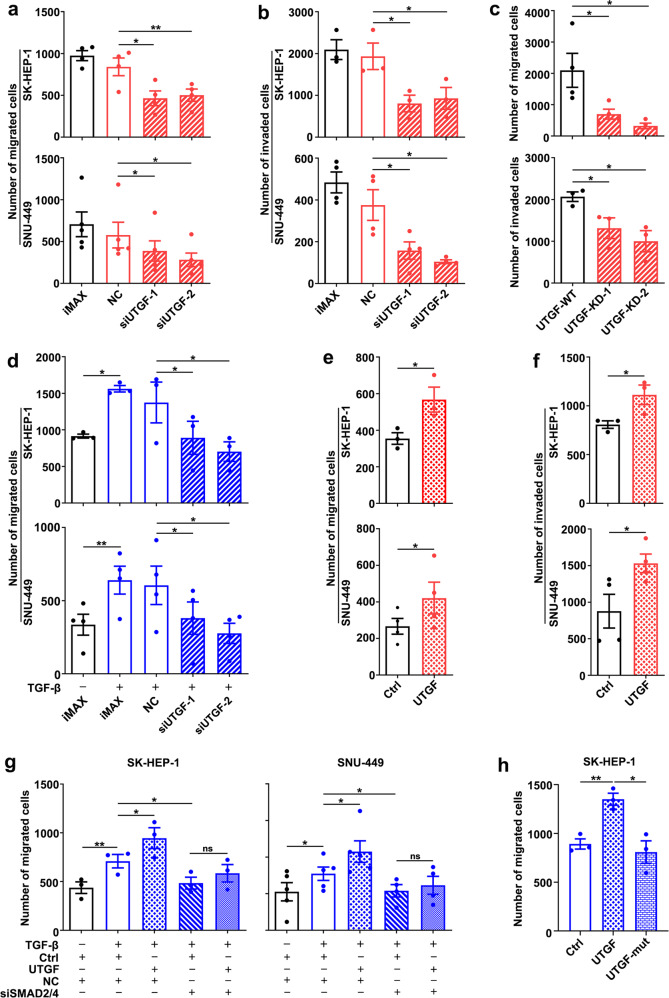
Lnc-UTGF promotes migration and invasion of tumor cells by enhancing the TGF-β/SMAD signaling. **a**–**c** Silencing lnc-UTGF suppressed migration and invasion of hepatoma cells. Cells were transfected with the indicated RNA duplexes for 36 h (**a**–**b**), or two sublines with heterozygous knockout of lnc-UTGF (UTGF-KD-1 and -2) and their control line (UTGF-WT; **c**) were examined. **d** Silencing lnc-UTGF attenuated the TGF-β-stimulated migration of hepatoma cells. Cells that were transfected with the indicated RNA duplexes for 24 h were incubated without or with TGF-β for another 24 h. **e**–**f** Overexpressing lnc-UTGF promoted migration and invasion of hepatoma cells. Cells stably expressing lnc-UTGF and its control cells (Ctrl) were examined. **g**–**h** Silencing of SMAD2/4 or mutation of the SMAD2/4-binding sites in lnc-UTGF attenuated the pro-migration effect of lnc-UTGF. For **g**, cells stably expressing lnc-UTGF and its control cells (Ctrl) were transfected with the indicated RNA duplexes for 24 h, then incubated without or with TGF-β for 24 h. For **h**, cells stably expressing full-length lnc-UTGF with wild-type sequence or with mutant SMAD2/4-binding sequences (UTGF-mut) were incubated with TGF-β for 24 h. For **a**–**h**, cells were added to transwell chambers without or with Matrigel coatings and incubated for 10 h, followed by staining with crystal violet. All the migrated/invaded cells were counted. siSMAD2/4, cells transfected with the mixture of siRNAs targeting SMAD2 and SMAD4. + or −, cells with (+) or without (−) the indicated treatment. Error bars: SEM from three independent experiments. *, *P* < 0.05; **, *P* < 0.01; ns, not significant

Given that the TGF-β/SMAD signaling plays essential roles in cell proliferation, apoptosis, and metastasis, the function of the TGF-β/SMAD-lnc-UTGF positive feedback loop was elucidated. As shown, neither silencing lnc-UTGF nor overexpressing lnc-UTGF affected cell viability or apoptosis (Supplementary Fig. S11a–d). Moreover, lnc-UTGF did not influence the colony formation of hepatoma cells (Supplementary Fig. S11e, f), indicating that lnc-UTGF may not significantly affect tumor cell growth. Subsequent analysis detected lnc-UTGF at a low level in cell lines with low metastatic potential (L02, HepG2) and at a higher level in those with high metastatic activity (SK-HEP-1, MHCC97H, SNU-449) (Supplementary Fig. S12). Further in vitro transwell assays revealed that the migration and invasion abilities were significantly inhibited in the hepatoma cells with siRNA targeting lnc-UTGF (Fig. 6a, b; Supplementary Fig. S13a, b), with heterozygous knockout (Fig. 6c; Supplementary Fig. S13c) or stable knockdown of lnc-UTGF (Supplementary Fig. S13d, e). And silencing lnc-UTGF also attenuated the TGF-β-stimulated migration of hepatoma cells (Fig. 6d; Supplementary Fig. S13f). On the other hand, ectopic expression of lnc-UTGF increased the migration and invasion of cells without or with TGF-β treatment (Fig. 6e–g, Supplementary Fig. S14a-c), whereas this promoting effect of lnc-UTGF was attenuated when SMAD2/4 were knocked down (Fig. 6g, Supplementary Fig. S14c). Notably, mutations in the SMAD2/4-binding sites abrogated the role of lnc-UTGF in promoting migration of hepatoma cells (Fig. 6h, Supplementary Fig. S14d), suggesting that lnc-UTGF may promote migration/invasion via SMAD2/4. We then analyzed the well-recognized TGF-β downstream genes involved in the regulation of cell proliferation (c-Myc, CDKN1A), apoptosis (BIM and DAPK), and metastasis (SNAIL1, MMP2). In agreement with the phenotype observations, TGF-β or siUTGF did not affect the mRNA levels of c-Myc, CDKN1A, BIM, and DAPK in our cell models (Supplementary Fig. S15a, b), whereas the expressions of SNAIL1 and MMP2 were significantly enhanced upon TGF-β stimulation, and this stimulatory effect was attenuated by knocking down lnc-UTGF (Supplementary Fig. S15c). Consistently, ectopic expression of lnc-UTGF increased the mRNA levels of SNAIL1 and MMP2 (Supplementary Fig. S15d).

**Fig. 7:**
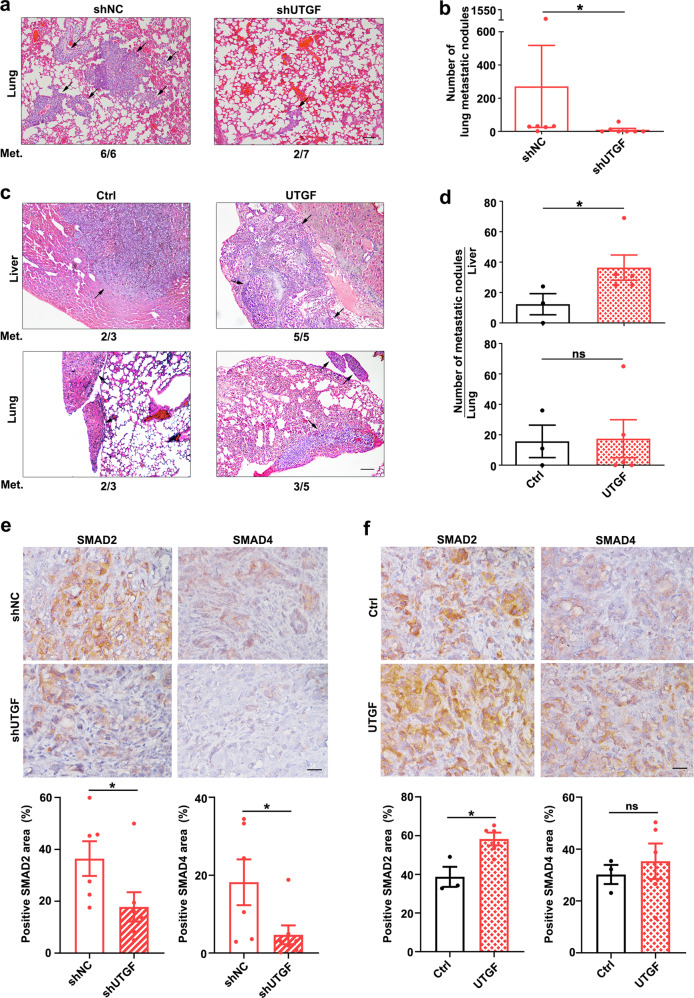
Lnc-UTGF promotes in vivo metastasis in a mouse xenograft model. **a**, **b** Xenografts of stable lnc-UTGF-silencing cells displayed a lower rate of lung metastasis and fewer metastatic nodules in the lung. **c**, **d** Xenografts of stable lnc-UTGF-overexpressing cells displayed a higher rate of liver metastasis and more metastatic nodules in the liver. For **a**–**d**, SK-shNC (shNC, *n* = 6) and SK-shUTGF (shUTGF, *n* = 7) or SK-Ctrl (Ctrl, *n* = 3) and SK-UTGF (UTGF, *n* = 5) sublines were implanted into the liver of BALB/c nude mice. Hematoxylin-eosin staining was performed on serial sections of lungs and livers to detect the metastatic nodules (**a**, **c**). The number of metastatic nodules is shown (**b**, **d**). Met. metastasis rate. Scale bar, 50 µm. **e** Silencing lnc-UTGF decreased the protein levels of SMAD2 and SMAD4 in mouse xenografts. **f** Ectopic expression of lnc-UTGF increased SMAD2 and SMAD4 levels in mouse xenografts. For **e**–**f**, the protein levels of SMAD2 and SMAD4 in mouse xenografts were detected by immunohistochemistry staining. Representative images (*upper panels*) and quantitative data (*lower panels*) are shown. Scale bar, 25 µm. Error bar, SEM. *, *P* < 0.05; **, *P* < 0.01. ns, not significant

We next demonstrated the effect of lnc-UTGF on tumor metastasis in vivo using mouse models. Compared with shNC group, the xenografts derived from lnc-UTGF-silencing cells displayed a lower rate of lung metastasis (Fig. 7a, shNC vs. shUTGF: 6/6 vs. 2/7), and also showed fewer metastatic nodules in the lung (Fig. 7b). No liver metastasis was observed in shNC and shUTGF group. Further gain-of-function studies showed that compared with the control group, the xenografts derived from lnc-UTGF-overexpressing cells had a higher rate of liver metastasis (Fig. 7c, *upper panel*, Ctrl vs. UTGF: 2/3 vs. 5/5) and more metastatic nodules in the liver (Fig. 7d, *upper panel*), although overexpressing lnc-UTGF did not further increase lung metastasis (Fig. 7c, *lower panel*, Ctrl vs. UTGF: 2/3 vs. 3/5; Fig. 7d, *lower panel*). Consistent with the in vitro findings, the protein levels of SMAD2 and SMAD4 were significantly reduced in lnc-UTGF-silencing xenografts but were increased in lnc-UTGF-overexpressing tumors (Fig. 7e, f). Neither silencing nor overexpressing lnc-UTGF affected tumor growth and proliferation signal in xenografts (Supplementary Fig. S16a-f). These findings suggest that lnc-UTGF may enhance TGF-β signaling and in turn promote metastasis by increasing SMAD2/4 levels.

**Fig. 8:**
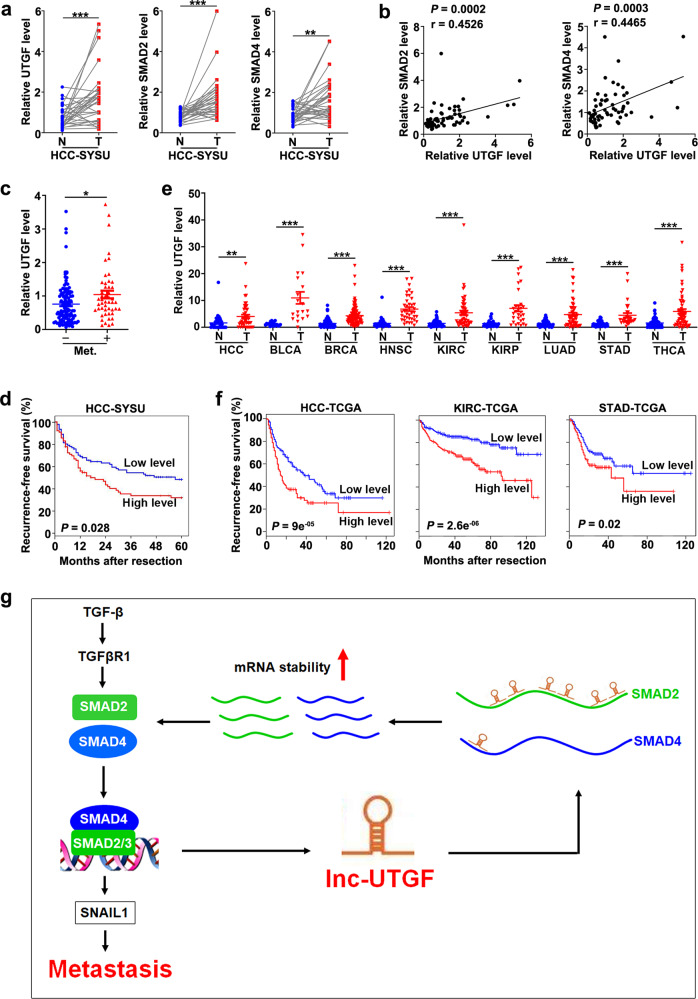
Lnc-UTGF is upregulated in human tumor tissues and correlated with a worse prognosis. **a** Lnc-UTGF, SMAD2, and SMAD4 were upregulated in HCC tissues. **b** The lnc-UTGF level was positively correlated with mRNA levels of SMAD2 and SMAD4. For **a**–**b**, the levels of lnc-UTGF, SMAD2, and SMAD4 were examined in 31 paired HCCs and adjacent non-tumor liver tissues by qPCR analysis. RNA levels in HCC tissue relative to that in matched adjacent non-tumor tissue (T/N) were used for Pearson’s correlation coefficient analysis in **b**. HCC-SYSU, HCC samples from Sun Yat-sen University. **c**–**d** A higher lnc-UTGF level was significantly associated with higher metastasis potential and worse recurrence-free survival. Lnc-UTGF levels were detected in 162 HCC tissues by qPCR analysis. For **c**, “−” indicates patients without metastasis potential (*n* = 113); “+” indicates patients with metastasis potential (Met.) (*n* = 49), including HCC patients who had tumor thrombus in the veins of adjacent non-tumor liver or in the portal vein at the time of surgery or who had relapse with lymph node metastasis or extrahepatic metastasis within 5 years post-operation. For **d**, the 54^th^ percentile of the lnc-UTGF level in 162 HCC tissues was chosen as the cut-off value for separating the lnc-UTGF-low level group (*n* = 88) from the lnc-UTGF-high level group (*n* = 74). **e**–**f** Lnc-UTGF was upregulated in various types of tumors and was associated with a worse recurrence-free survival. Lnc-UTGF levels in nine types of paired tumor tissues and adjacent non-tumor tissues were analyzed using datasets from The Cancer Genome Atlas (TCGA). BLCA, bladder urothelial carcinoma. BRCA, breast invasive carcinoma. HNSC, head and neck squamous cell carcinoma. KIRC, kidney renal clear cell carcinoma. KIRP, kidney renal papillary cell carcinoma. LUAD, lung adenocarcinoma. STAD, stomach adenocarcinoma. THCA, thyroid carcinoma. HCC/KIRC/STAD-TCGA, HCC/KIRC/STAD samples from TCGA datasets. U6 was used as an internal control. Error bar, SEM. *, *P* < 0.05; **, *P* < 0.01; ***, *P* < 0.001. **g** Schematic illustration depicting the TGF-β/SMAD/lnc-UTGF positive feedback loop and its role in tumor metastasis

We further validated the pro-metastasis function of lnc-UTGF in human samples. As shown, the levels of lnc-UTGF, SMAD2, and SMAD4 were significantly higher in HCC tissues compared with the matched adjacent non-tumor liver tissues (Fig. 8a). And the lnc-UTGF level was positively correlated with mRNA levels of SMAD2, SMAD4, and SNAIL1 (Fig. 8b; Supplementary Fig. S17). Furthermore, a higher lnc-UTGF level in HCC tissues was significantly associated with higher metastasis potential (Fig. 8c), higher TNM stages (Supplementary Table S1), and worse recurrence-free survival (Fig. 8d). Interestingly, transcriptome data derived from the Genotype-Tissue Expression (GTEx) database showed that lnc-UTGF was universally expressed in different tissues (Supplementary Fig. S18). Based on the TCGA database, lnc-UTGF was upregulated in various types of tumors (Fig. 8e) and was associated with poorer recurrence-free survival of patients (Fig. 8f), suggesting that lnc-UTGF may play a universal role in tumor development.

In summary, we disclose that upon TGF-β stimulation, SMAD2/3 directly binds to the lnc-UTGF promoter and induces lnc-UTGF transcription, whereas lnc-UTGF in turn increases the stability of SMAD2/4 mRNAs via a direct interaction, which consequently promotes the TGF-β/SMAD signaling and thus tumor metastasis (Fig. 8g).

## Discussion

The positive and negative feedback loops play critical roles in physiological processes by amplifying and terminating the signaling, respectively. Deregulation of feedback loops contributes to abnormal signaling transduction and subsequent disease development. Aberrant activation of the TGF-β/SMAD signaling pathway facilitates tumor metastasis and is often observed in HCC.^20–26^ Whether lncRNA is involved in the feedback loop of the TGF-β signaling in HCC remains unreported. In the present report, we found that TGF-β treatment activated lnc-UTGF transcription via SMAD2/3, and lnc-UTGF in turn enhanced the TGF-β signaling by increasing the stability of SMAD2/4-mRNAs, which consequently promoted tumor metastasis.

Recent studies have revealed that the levels of four lncRNAs, including lnc-ATB, PVT1, HCCL5, and H19, are affected by TGF-β in HCC cells. However, how TGF-β modulates the levels of lnc-ATB and PVT1 is unexplored, whereas TGF-β indirectly upregulates HCCL5 and reduces H19 via ZEB1 and SOX2, respectively.^27–30^ Here, we demonstrated that lnc-UTGF expression was transcriptionally induced by the canonical TGFβR1/SMAD signaling, based on the following evidence: (1) TGF-β increased lnc-UTGF expression in a time- and dose-dependent manner; (2) Inhibition of TGFβR1 or silencing of SMAD2/3/4 suppressed the TGF-β-induced lnc-UTGF transcription; (3) Deletion or mutation of putative SBEs in the lnc-UTGF promoter abrogated the role of TGF-β in enhancing the activity of lnc-UTGF promoter; (4) EMSA, antibody supershift and ChIP assays revealed that SMAD2/3 directly interacted with the lnc-UTGF promoter in vitro and in vivo. These data disclose a novel TGF-β/SMAD-transactivated lncRNA.

The SMAD7-mediated negative feedback loop is essential in preventing abnormal activation of the TGF-β signaling and its dysfunction facilitates cancer metastasis.^5,23,31,32^ SMAD7 is usually downregulated in tumor cells by extracellular and intracellular perturbations, such as UV irradiation, inflammation, and dysregulation of miRNAs targeting SMAD7.^31,33–36^ Here, we identified a novel positive feedback loop that was mediated by lncRNA and showed that lnc-UTGF could promote the TGF-β/SMAD signaling and in turn hepatoma metastasis. We further revealed that upregulation of lnc-UTGF occurred in various types of human cancers and was associated with higher metastasis potential and worse recurrence-free survival, suggesting that upregulation of lnc-UTGF may represent a universal mechanism that amplifies the TGF-β signaling via the positive feedback loop in cancer cells.

Previous studies have shown that the levels of SMAD2, SMAD3, and SMAD4 are significantly increased in HCC,^22,37–40^ although the underlying mechanism remains unclear. To date, only two lncRNAs have been reported to regulate SMADs’ expression. Mondal et al. showed that maternally expressed gene 3 (MEG3) bound to the promoter-distal GA-rich sequences of SMAD2 and then recruited EZH2 to suppress SMAD2 transcription.^41^ Wu et al. found that LINC00941 competed with β-TrCP to bind the MH2 domain of SMAD4 protein, and thus prevented the degradation of SMAD4 protein.^42^ Here, we found that lnc-UTGF directly interacted with the mRNAs of SMAD2 and SMAD4 via highly complementary base-pairing, and thus stabilized their mRNAs, which disclose a new mechanism by which lncRNA regulates the levels of SMADs.

Recently, Liang et al. showed that lnc-UTGF promoted EMT in serous ovarian cancer by competitively binding miR-101-3p and then increasing ZEB1 expression.^43^ However, we found that lnc-UTGF was unable to increase the ZEB1 level in the hepatoma cells we studied (data not shown). Instead, we revealed that lnc-UTGF increased the expression of SNAIL1, which is an important EMT inducer. Notably, lnc-UTGF promoted the TGF-β-stimulated migration of hepatoma cells, whereas this promoting effect was attenuated when SMAD2 and SMAD4 were knocked down. Moreover, mutations in the SMAD2/4-binding sites abrogated the pro-migration effect of lnc-UTGF, suggesting that the pro-metastasis ability of lnc-UTGF in hepatoma cells mostly depends on its effect in increasing SMAD2/4 levels and enhancing the TGF-β/SMAD signaling.

In summary, we identify a novel TGF-β/SMAD/lnc-UTGF positive feedback circuitry and disclose that upregulation of lnc-UTGF augments the TGF-β/SMAD signaling via this feedback loop and thereby promotes HCC metastasis. These findings highlight the importance of lncRNA in regulating the TGF-β signaling and HCC metastasis, which may be exploited for anti-metastasis therapy.

## Materials and methods

Additional information is provided in Supplementary Material and Methods.

### Reagents

The following reagents were used: recombinant TGF-β1 (240-B-002, R&D Systems, Minneapolis, MN, USA), actinomycin-D (ActD, 15021 S, Cell Signaling Technology, CST, Beverly, MA, USA), SB525334 (S1476, Selleckchem, Houston, TX, USA). Unless otherwise indicated, a final concentration of 2 ng/ml TGF-β1, 5 μg/ml ActD, or 2 μM SB525334 was used.

### RNA oligoribonucleotides

Small interference RNAs (siRNAs) targeting human lnc-UTGF (Ensembl transcript ID: ENST00000428667.1), TGFβR1 (ENST00000374994.9), SMAD2 (ENST00000262160.11), SMAD3 (ENST00000327367.9), and SMAD4 (ENST00000342988.7) transcripts are designated as siUTGF, siTGFβR1, siSMAD2, siSMAD3, and siSMAD4, respectively, and were purchased from GenePharma (Shanghai, China). The negative control (NC) RNA duplex for siRNAs is non-homologous to any human genome sequence. The sequences of RNA duplexes are provided in Supplementary Table S2.

### Rapid-amplification of cDNA ends (RACE)

The 5′- and 3′-end of lnc-UTGF transcript was determined by the 5′RACE (D315, TaKaRa, Kyoto, Japan) and 3′RACE System (Version 2.0; Invitrogen, Carlsbad, CA, USA) respectively, using total RNA from normal liver tissues.

### Isolation of cytoplasmic and nuclear RNA

Nuclear and cytoplasmic fractions were separated using NE-PER Nuclear and Cytoplasmic Extraction Reagent kit (Pierce, Rockford, IL, USA) according to the manufacturer’s instructions.

### Plasmid construction

Lentivirus expression vectors pCDH-UTGF, pCDH-UTGF-mut, pCDH-S1m-UTGF, pCDH-shNC, and pCDH-shUTGF were generated using pCDH-CMV-MCS-EF1-copGFP-T2A-Puro (System Biosciences, Palo Alto, CA, USA), which contained a copGFP expression cassette and was designated pCDH-Ctrl in this study. The pXPR_001-dual-proUTGF was produced based on the lentiviral CRISPR plasmid pXPR_001 vector (Addgene, Boston, MA, USA). pc3-UTGF, pc3-UTGF-antisense, and GFP-fusion protein expression vectors (pc3-ORF-GFP and pc3-GAPDH-GFP) were generated using pcDNA3.0 (Invitrogen). Firefly luciferase reporter vectors pGL3-basic-p(−1.6/+0.1k), pGL3-basic-p(−1.2/+0.1k), pGL3-basic-p(−0.8/+0.1k), pGL3-basic-p(−0.4/+0.1k), pGL3-basic-p(−0.1/+0.1k), pGL3-basic-p(mutSBE), and pGL3-basic-p(delSBE) were constructed based on pGL3-basic vector (Promega, Madison, WI, USA).

### Lentivirus production and infection

For lentivirus production, HEK293T cells were co-transfected with the lentivirus expression vector that contained the target sequence and the packaging plasmid mix (Lenti-X HTX Packaging Mix, Clontech, Palo Alto, CA, USA) via calcium phosphate precipitation. The lentivirus supernatant was harvested and stored in aliquots at −80 °C until use. Target cells, grown to 30% confluence at 24-well plate, were incubated in 1 ml lentivirus supernatant supplemented with 10 μg/ml polybrene (Millipore, Billerica, MA, USA).

### Cell lines

HEK293T cells and hepatoma cell line SK-HEP-1 were cultured in Dulbecco’s modified Eagle’s medium (DMEM, Gibco, ThermoFisher Scientific, Waltham, Massachusetts, USA) supplemented with 10% fetal bovine serum (FBS, Gibco). Another hepatoma cell line SNU-449 was maintained in RPMI 1640 medium (Gibco) supplemented with 10% FBS (Gibco). All cells were cultured in a humidified atmosphere of 5% CO_2_ at 37 °C.

The stable cell lines were established by infecting SK-HEP-1 or SNU-449 cells with lentivirus that expressed the target sequence. Sublines with stable expression of lnc-UTGF with wild-type sequence (SK-UTGF, SNU-UTGF) or with mutant SMAD2- and SMAD4-binding sequences (SK-UTGF-mut), S1m-tagged lnc-UTGF (SK-S1m-UTGF) and the control lines (SK-Ctrl, SNU-Ctrl), as well as SK-HEP-1 cells with stable silencing (SK-shUTGF) or heterozygous knockout of lnc-UTGF (SK-UTGF-KD-1, SK-UTGF-KD-2) and the matched control line SK-shNC or SK-UTGF-WT, were constructed.

### Cell transfection

RNA oligos were reversely transfected using Lipofectamine RNAiMAX (Invitrogen). A final concentration of 50 nM RNA duplexes was used. Transfection of plasmid DNA alone or together with RNA duplex was conducted using Lipofectamine 2000 (Invitrogen).

### Analysis of gene expression

The expression level of target genes was analyzed by real-time quantitative polymerase chain reaction (qPCR) and Western blotting.

### Luciferase reporter assay

Luciferase activity was measured using the dual-luciferase reporter assay system (Promega). *Renilla* luciferase expressed by pRL-CMV (Promega) was used as a control to correct the difference in both transfection and harvest efficiency.

To characterize the lnc-UTGF promoter, cells were co-transfected with 4 ng pRL-CMV, 100 ng firefly luciferase reporter vector, and 50 nM RNA duplexes for 36 h, followed by incubation without or with 2 ng/ml TGF-β for another 12 h before the luciferase activity assay.

To examine the activity of TGF-β signaling, a luciferase reporter plasmid (pSBE, generously provided by Peter ten Dijke, Leiden University Medical Center, Leiden, The Netherlands) bearing twelve tandem SMAD-binding elements (SBEs) was used. Cells were transfected with 50 nM RNA duplexes for 12 h and then co-transfected with 100 ng pSBE and 4 ng pRL-CMV for 24 h, followed by incubation in 2 ng/ml TGF-β for 12 h before the luciferase activity assay.

### Electrophoretic mobility shift assay (EMSA)

EMSA and antibody-supershift assays were conducted as described previously.^44^ Briefly, the biotin-labeled probes were incubated with nuclear extracts of SK-HEP-1 cells at room temperature for 30 min and subjected to native-PAGE. For competition assay, nuclear extract was pre-incubated with 100-fold molar excess of unlabeled oligonucleotides prior to adding labeled probe. For antibody-supershift assay, nuclear extract was pre-incubated with anti-SMAD2/3 antibody or isotype-matched IgG before adding to the binding reaction solution that contained labeled probe. Detection of the biotinylated probe in blots was performed using Chemiluminescent EMSA Kit (Beyotime, Shanghai, China). The sequences of probes are listed in Supplementary Table S2.

### Chromatin Immunoprecipitation (ChIP) assay

SK-HEP-1 cells were treated with 2 ng/ml TGF-β for 2 h, and then cross-linked by formaldehyde. The chromatin complexes were immunoprecipitated using anti-SMAD2/3 antibody (cat.8685, CST), or isotype-matched IgG (negative control), then collected with Protein A/G MagBeads (Bimake, Houston, TX, USA). The immunoprecipitated DNAs were analyzed by semi-quantitative PCR or qPCR with primers listed in Supplementary Table S2.

### Immunofluorescence staining

Immunofluorescence staining assay was performed to examine the expression and localization of SMAD2 and SMAD4.

### S1m-tagged RNA affinity purification

RNA interacted with lnc-UTGF was identified by affinity purification via S1m-tag. S1m-UTGF and their binding RNAs were pulled down by streptavidin Dynabeads (65001, Invitrogen). The untagged lnc-UTGF was used as a negative control. RNA was extracted from the precipitates by TRIzol reagent (Invitrogen).

### RNA pull-down assay

RNA pull-down assay was performed using in vitro transcribed biotinylated RNA and streptavidin Dynabeads (Invitrogen). The retrieved RNAs were extracted by TRIzol and analyzed by qPCR with primers listed in Supplementary Table S2.

#### In vitro cell proliferation and apoptosis assays

Cell counting and colony formation assays were used to access the in vitro proliferation of tumor cells. Nuclear morphological examination by DAPI staining was used to evaluate the apoptosis of tumor cells.

#### In vitro migration and invasion assays

The migration and invasion of tumor cells were analyzed in 24-well Boyden chambers with 8-μm pore size polycarbonate membranes (Corning, NY, USA). For invasion assays, the membranes were coated with Matrigel (3432-005-01, R&D Systems) to form matrix barriers. Briefly, SK-HEP-1 or SNU-449 cells in serum-free DMEM or RPMI were placed into the upper chamber of 24-well Boyden chamber coated without or with Matrigel (R&D Systems), while the lower chamber was filled with 600 μl 10% FBS-containing DMEM/RPMI. After 10 h of incubation, cells were fixed and stained with crystal violet. All the migrated/invaded cells were counted.

### Mouse model studies

All procedures for animal experiments were performed in accordance with the Guide for the Care and Use of Laboratory Animals (National Institutes of Health publication no. 80-23, revised 1996) and according to the Sun Yat-sen University Institutional Ethical Guidelines for animal experiments.

SK-shNC, SK-shUTGF, SK-Ctrl, or SK-UTGF cells were resuspended in Matrigel (R&D Systems) and then inoculated under the capsule of the left hepatic lobe of male BALB/c nude mice at 5 weeks of age. Four weeks later, the xenografted mice were then applied to evaluate the metastasis. The length (*L*) and width (*W*) of the dissected tumors were measured with calipers and the tumor volume (*V*) was calculated using the formula *V* = (*L* × *W*^2^) × 0.5. Aliquots of tumor tissues were freshly frozen in liquid nitrogen, or fixed in 10% formalin, and embedded in paraffin. To evaluate the metastasis, serial sections from lungs and livers were stained with hematoxylin-eosin (HE) and screened for metastatic nodules.

### Human tissue specimens

Human HCC tissues were obtained from 162 patients who underwent HCC resection and were pathologically confirmed as hepatocellular carcinoma at the Cancer Center of Sun Yat-sen University. Adjacent non-tumor liver tissues with a distance of 1.5–3 cm from the tumor tissues were collected. The patients had not received any local or systemic anti-cancer treatments prior to the surgery, and no postoperative anti-cancer therapies were administered prior to relapse. All patients were followed postoperatively to assess survival rates and to monitor for recurrence and metastases. The relevant characteristics of the studied subjects are shown in Supplementary Table S1. Informed consent was obtained from each patient, and the study was approved by the Institute Research Ethics Committee at the Cancer Center.

### Statistical analysis

The differences in gene expression levels between the paired HCC tissues and adjacent non-tumor liver tissues were analyzed by paired *t*-test. The correlations between the RNA levels of different genes in HCC tissues were explored with Pearson’s correlation coefficient. Recurrence-free survival was calculated from the date of HCC resection to the time of first recurrence or death. Patients who were lost to follow-up were treated as censored events. Chi-square test analysis and Kaplan–Meier survival curves were performed using SPSS version 13.0 (SPSS Inc., Chicago, IL).

Data were expressed as the mean ± standard error of the mean (SEM) from at least three independent experiments. Unless indicated, Student’s *t* test was performed to compare the differences between two groups and one-way ANOVA was applied to compare more than two groups. All statistical tests were two-sided and *P* < 0.05 was considered to be statistically significant. All analyses were performed using GraphPad Prism version 8.0 (GraphPad Software, Inc., San Diego, CA, USA).

## Supplementary information

Supporting information

## Data Availability

The data that support this study are present in the manuscript and supplementary information, and are available from the corresponding author upon request. The RNA-seq data analyzed during the current study are accessed with the Gene Expression Omnibus (GEO) series accession numbers: GSE108554, GSE98225, and GSE101809.
