# Correction: Identification of a TGF-β/SMAD/lnc-UTGF positive feedback loop and its role in hepatoma metastasis

**DOI:** 10.1038/s41392-021-00839-2

**Published:** 2021-12-27

**Authors:** Meng-Zhi Wu, Yi-chuan Yuan, Bi-Yu Huang, Jin-Xi Chen, Bin-Kui Li, Jian-Hong Fang, Shi-Mei Zhuang

**Affiliations:** 1 MOE Key Laboratory of Gene Function and Regulation, School of Life Sciences, Collaborative Innovation Center for Cancer Medicine, Sun Yat-sen University, Xin Gang Xi Road 135#, Guangzhou, 510275 P. R. China; 2 Department of Hepatobiliary Surgery, Sun Yat-sen University Cancer Center, Dong Feng Road East 651#, Guangzhou, 510060 P. R. China

**Keywords:** Metastasis, Non-coding RNAs

Correction to: *Signal Transduction and Targeted Therapy* 10.1038/s41392-021-00781-3, published online 17 November 2021

In this article^1^ Jian-Hong Fang should have been denoted as a corresponding author along with Shi-Mei Zhuang, but was inadvertently removed during the production process.

The original article has been corrected.
