# Supplementary material for: Identification of a TGF-β/SMAD/lnc-UTGF positive feedback loop and its role in hepatoma metastasis
Source: Signal Transduct Target Ther. 2021 Nov 17;6:395. doi: 10.1038/s41392-021-00781-3 (PMC8595887; doi:10.1038/s41392-021-00781-3)
Supplement: Supplementary file 1 — Supporting information [file 41392_2021_781_MOESM1_ESM.pdf]

Supplementary Materials for

Identification of a TGF-β/SMAD/lnc-UTGF feedback loop and its role in hepatoma metastasis

Meng-Zhi Wu, Yi-chuan Yuan, Bi-Yu Huang, Jin-Xi Chen, Bin-Kui Li, Jian-Hong Fang and Shi-Mei Zhuang

Correspondence to: Prof. Shi-Mei Zhuang (zhuangshimei@163.com or LSSZSM@mail.sysu.edu.cn), Dr. Jian-Hong Fang (fangjh6@mail.sysu.edu.cn.)

This PDF file includes:

Supplementary Materials and Methods

Supplementary Figure S1 to S18

Supplementary Table S1 to S2

Supplementary Materials and Methods

Rapid amplification of cDNA ends (RACE)

The 5′-end of the lnc-UTGF transcript was characterized using a 5′-Full RACE Kit (D315, TaKaRa, Kyoto, Japan). Total RNAs from normal liver tissues were subjected to reverse transcription with random 6-mers. The tailed cDNAs were then amplified using GSP1 and 5′-RACE outer primer, followed by a second PCR round with GSP2 and 5′-RACE inner primer.

The 3′-end of lnc-UTGF transcript was determined using a 3′RACE System (Invitrogen, Carlsbad, CA, USA). Total RNAs from normal liver tissues were subjected to reverse transcription with a 3′RACE-adaptor primer, followed by nested PCR using gene-specific primers (GSP3 and GSP4) and abridged universal amplification primer (AUAP).

The sequences of the PCR-amplified 5′- and 3′-end fragments were confirmed by direct sequencing. The sequences of DNA oligonucleotides are provided in Supplementary Table S2

Plasmid construction

The following expression vectors were used: lentivirus expression vectors (pCDH-UTGF, pCDH-UTGF-mut, pCDH-S1m-UTGF, pCDH-shNC, pCDH-shUTGF, and pXPR_001-dual-proUTGF); pc3-UTGF and pc3-UTGF-AS expression vectors; GFP-fusion protein expression vectors (pc3-ORF-GFP and pc3-GAPDH-GFP); firefly luciferase reporter vectors (pGL3-basic-p(-1.6/+0.1k), pGL3-basic-p(-1.2/+0.1k), pGL3-basic-p(-0.8/+0.1k), pGL3-basic-p(-0.4/+0.1k), pGL3-basic-p(-0.1/+0.1k), pGL3-basic-p(mutSBE) and pGL3-basic-p(delSBE)).

To produce pCDH-UTGF and pCDH-UTGF-mut vectors, full-length lnc-UTGF with wild-type sequence or with mutant SMAD2/4-binding sequences was inserted into the *Eco*RI/*BamH*I sites of pCDH-CMV-MCS-EF1-CopGFP-T2A-Puro (System Biosciences, Palo

Alto, CA, USA), which contained a copGFP expression cassette and was designated as pCDH-Ctrl in this study. pCDH-S1m-UTGF was produced by inserting full-length lnc-UTGF into the *Eco*RI/*Swa*I sites of pCDH-S1m-vector. The backbone plasmid pCDH-S1m-vector was produced by inserting four tandem S1m sequences[1] that encoded the streptavidin-binding RNA aptamer into the *Xba*I/*Eco*RI sites of pCDH-Ctrl.

To construct pCDH-shNC and pCDH-shUTGF plasmids, the siRNA sequences (shNC: 5′-TGAATTAGATGGCGATGTT-3′; shUTGF: 5′-GCAAGGTTTATTCCTGGAT-3′), the spacer sequence (5′-CTCGAG) and the flanking *Eco*RI and *BamH*I sites were chemically synthesized, annealed and then inserted into the *Eco*RI/*BamH*I sites in the pCDH-U6 vector. pCDH-U6 was produced by replacing the CMV promoter with the U6 promoter.

To create pXPR_001-dual-proUTGF vector, two DNA oligonucleotides that encoded the guide RNAs targeting the -2000 ~ +200-bp sequence of lnc-UTGF, together with a sgRNA scaffold sequence and full-length sequence of the H1 promoter were inserted into the *BsmB*I site of the pXPR_001 vector (Addgene, Boston, MA, USA).

The pc3-UTGF and pc3-UTGF-AS were generated by respectively cloning full-length lnc-UTGF sequence or antisense sequence of lnc-UTGF into the *Eco*RI/*BamH*I sites of pcDNA3.0 (Invitrogen, Carlsbad, CA, USA). To create pc3-ORF-GFP, the predicted ORF with 5′-UTR of lnc-UTGF was fused in-frame to the N-terminus of GFP coding sequence (without ATG) and cloning into the *Eco*RI/*Xho*I sites of pcDNA3.0 (Invitrogen). The pc3-GAPDH-GFP plasmid that expressed GAPDH-GFP fusion protein was used as a positive control.

To identify the promoter of lnc-UTGF, the firefly luciferase reporter vectors pGL3-basic-p(-1.6/+0.1k) was generated by cloning the -1592 ~ +99-bp sequence of lnc-UTGF (chr21: 37801008 ~ 37802698) into the *Nhe*I/*Hind*III sites upstream of the firefly

luciferase gene in a pGL3-basic vector (Promega, Madison, WI, USA). The constructs with 5′-deletion of the lnc-UTGF promoter were generated by fusion PCR based on pGL3-basic-p(-1.6/+0.1k). The pGL3-basic-p(mutSBE) and pGL3-basic-p(delSBE) vectors that contained mutation or deletion of the predicted SMAD-binding elements (SBEs) in the lnc-UTGF promoter were generated by fusion PCR based on pGL3-basic-p(-0.4/+0.1k).

The sequences of all plasmids were confirmed by direct sequencing. The sequences of DNA oligonucleotides are provided in Supplementary Table S2.

Analysis of gene expression

Real-time quantitative polymerase chain reaction (qPCR) assay was performed to evaluate the RNA levels. Total RNAs were extracted using TRIzol reagent (Invitrogen) and reversely transcribed using M-MLV reverse transcriptase (M1701, Promega). qPCR was performed on a LightCycler 480 (Roche Diagnostics, Germany) using 2×SYBR Green qPCR Master Mix (B21202, Bimake, Houston, TX, USA). All reactions were performed in duplicate. The cycle threshold (Ct) values differed by less than 0.5 between duplicate wells. The level of target gene was normalized to that of U6, which yielded a $2^{-\Delta Ct}$ value. The sequences of qPCR primers are listed in Supplementary Table S2.

Western blotting was performed to determine the protein levels. The antibodies used included mouse polyclonal antibody against GAPDH (BM1623, Boster, Wuhan, China); rabbit monoclonal antibodies (mAb) against SMAD2 (cat.5339, Cell Signaling Technology, CST, Beverly, MA, USA), Ser465/467-phosphorylated SMAD2 (cat.18338, CST), SMAD3 (cat.9523, CST), Ser423/425-phosphorylated SMAD3 (cat.9520, CST) and SMAD4 (cat.46535, CST); rabbit polyclonal antibodies against TGFβR1 (cat.PB1154, Boster) and SMAD7 (cat.D160746, Sangon Biotech, Shanghai, China).

Immunofluorescence staining

Cells were fixed with 4% paraformaldehyde for 15 minutes, washed twice with pre-cooling PBS, permeabilized with 0.25% Triton X-100, blocked with 1% bovine serum albumin in PBS for 30 minutes at room temperature, followed by sequential incubation with rabbit mAbs against SMAD2 (cat.5339, CST) or SMAD4 (cat.46535, CST) at 4°C overnight and then with goat anti-rabbit Alexa Fluor 488 secondary antibody (A11008, Thermo Fisher Scientific, Waltham, MA, USA) at room temperature for 1 hour in the dark. The cell nuclei were stained with 1 μg/mL 4'-6'-diamidino-2-phenylindole (DAPI; Sigma-Aldrich). Samples were analyzed using a confocal microscope (LSM 880; Carl Zeiss, Oberkochen, Germany).

S1m-tagged RNA affinity purification

This experiment was carried out as previously described with modifications.[2] Briefly, ~2×10^7 SK-HEP-1 sublines with stable expression of lnc-UTGF or S1m-lnc-UTGF were washed with 1×PBS, pelleted by centrifugation at 500×g for 5 minutes, resuspended in 8 mL 1×PBS containing 0.37% formaldehyde and rotated at RT for 5 minutes, followed by the addition of glycine to a final concentration of 0.25 M to stop the crosslinking reaction. After two washes with 10 mL ice-cold 1×PBS, cell pellets were resuspended in ice-cold lysis buffer (150 mM NaCl, 10 mM HEPES at pH 7.4, 3 mM $MgCl_2$, 10% glycerol, 1% NP-40, 2 mM DTT, 1 mM PMSF, protease inhibitor cocktail, and 500 U/mL RNase inhibitor), incubated on ice for 10 minutes, and then sonicated 4 times with a Bioruptor. The cell lysates were centrifuged at 18,000×g, 4 °C for 10 minutes to remove insoluble materials, then precleared with 40 μL avidin agarose beads (20219, Pierce, Rockford, IL, USA) at 4 °C for 1 hour, followed by addition of 30 μL streptavidin Dynabeads (65001, Invitrogen) and then rotation at 4 °C for 3 hours to pull-down S1m-tagged RNA-RNA complexes. The beads were washed 5 times with ice-cold washing buffer (50 mM HEPES at pH 7.4, 400 mM NaCl, 0.1% TritonX-100, 10% glycerol, 2% NP-40, 1 mM EDTA, 1 mM PMSF, and 1 mM DTT) by rotation at 4 °C for 5 minutes each time, followed by addition of 100 μL elution buffer (50 mM HEPES at pH 7.4,

5 mM EDTA, 100 mM NaCl, 1% SDS, and 10 mM DTT) and heating at 70 °C for 45 minutes to reverse the formaldehyde crosslinking. RNAs were extracted from the precipitates by TRIzol reagent (Invitrogen) and detected by qPCR.

RNA pull-down assay

RNA pull-down was performed as described previously with modifications.[3] Briefly, biotin-labelled RNAs were transcribed *in vitro* from PCR product with Biotin RNA Labeling Mix (Roche) and T7 RNA polymerase (Roche), treated with RNase-free DNase I (ThermoFisher Scientific, Waltham, Massachusetts, USA) and purified by LiCl precipitation. SK-HEP-1 cells (~ 2×10^7) were lysed in 1 mL IP-lysis buffer (Pierce). Cell lysates were centrifuged at 18,000×g, 4 °C for 10 minutes, and the supernatants were then mixed with 3 μg of renatured biotin-labelled RNAs by rotation at RT for 1 hour, followed by addition of 30 μL streptavidin Dynabeads (Invitrogen) and incubation at RT for another 0.5 hours to pull-down the biotin-labelled RNA-RNA complexes. The beads were washed with IP-lysis buffer 5 times, followed by the addition of 100 μL elution buffer (50 mM HEPES at pH 7.4, 5 mM EDTA, 100 mM NaCl, 1% SDS, and 10 mM DTT). The retrieved RNAs were then extracted by TRIzol reagent (Invitrogen) and detected by qPCR.

Cell counting assay

Cell counting assay was used to evaluate cell growth. Cells (1×10^4 SK-HEP-1, 2×10^4 SNU-449) were seeded in a 24-well plate and transfected with 50 nM siRNAs, or infected with pCDH-Ctrl or pCDH-UTGF lentivirus, and then cultured for 72 hours before cell counting by Countstar (ALIT Life Sciences, Shanghai, China).

Colony formation assay

The viable SK-HEP-1 or SNU-449 cells (300) infected with pCDH-Ctrl or pCDH-UTGF lentivirus were placed in a 6-well plate and maintained in complete medium for 10 days.

Colonies were fixed in methanol and stained with a 0.1% crystal violet solution in 20% methanol for 15 min.

Apoptosis analysis

Cell apoptosis was evaluated by morphological examination. Cells (1×10^4 SK-HEP-1, 1.5×10^4 SNU-449) were seeded in a 48-well plate and transfected with 50 nM siRNAs, or infected with pCDH-Ctrl or pCDH-UTGF lentivirus, and then cultured for 48 hours, followed by staining with DAPI (Sigma-Aldrich) and analysis under fluorescence microscopy. Those cells with fragmented or condensed nuclei were considered as apoptotic cells. At least 400 cells were examined for each sample.

References

1 Kallen, A. N. *et al.* The imprinted H19 lncRNA antagonizes let-7 microRNAs. *Molecular cell* **52**, 101-112 (2013).

2 Xie, C. *et al.* A hMTR4-PDIA3P1-miR-125/124-TRAF6 Regulatory Axis and Its Function in NF kappa B Signaling and Chemoresistance. *Hepatology (Baltimore, Md.)* **71**, 1660-1677 (2020).

3 Wang, Y. L. *et al.* Lnc-UCID Promotes G1/S Transition and Hepatoma Growth by Preventing DHX9-Mediated CDK6 Down-regulation. *Hepatology (Baltimore, Md.)* **70**, 259-275 (2019).

Supplementary Figures and Figure Legends

Supplementary Fig. S1

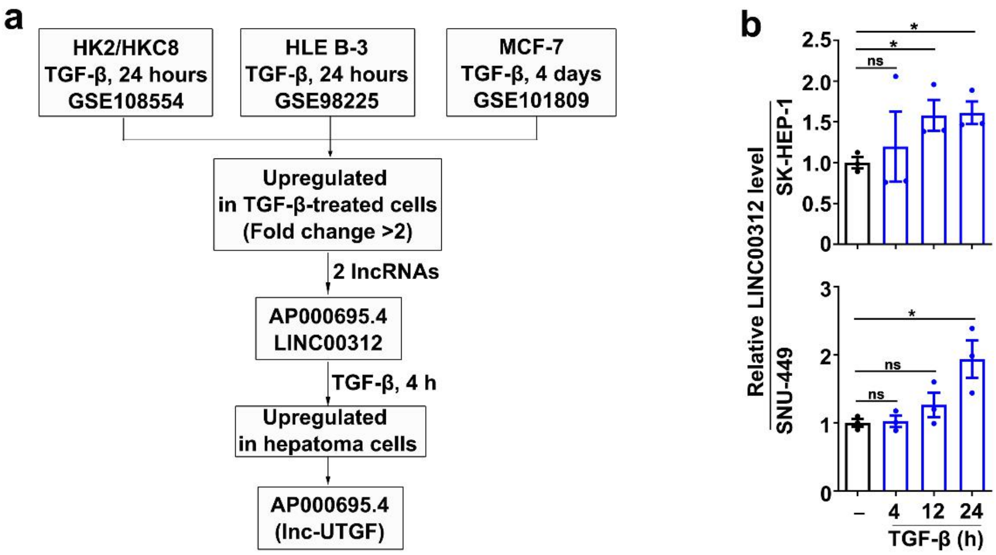

Supplementary Figure S1. Screening of TGF-β-induced lncRNAs. (a) Workflow of the screening procedure to identify the lncRNAs induced by TGF-β. **(b)** Effect of TGF-β treatment in LINC00312 expression. SK-HEP-1 or SNU-449 cells were untreated (−) or treated with TGF-β for the indicated time and then subjected to qPCR. U6 was used as an internal control. Error bar: SEM from at least three independent experiments. *, $P < 0.05$; ns, not significant.

Supplementary Fig. S2

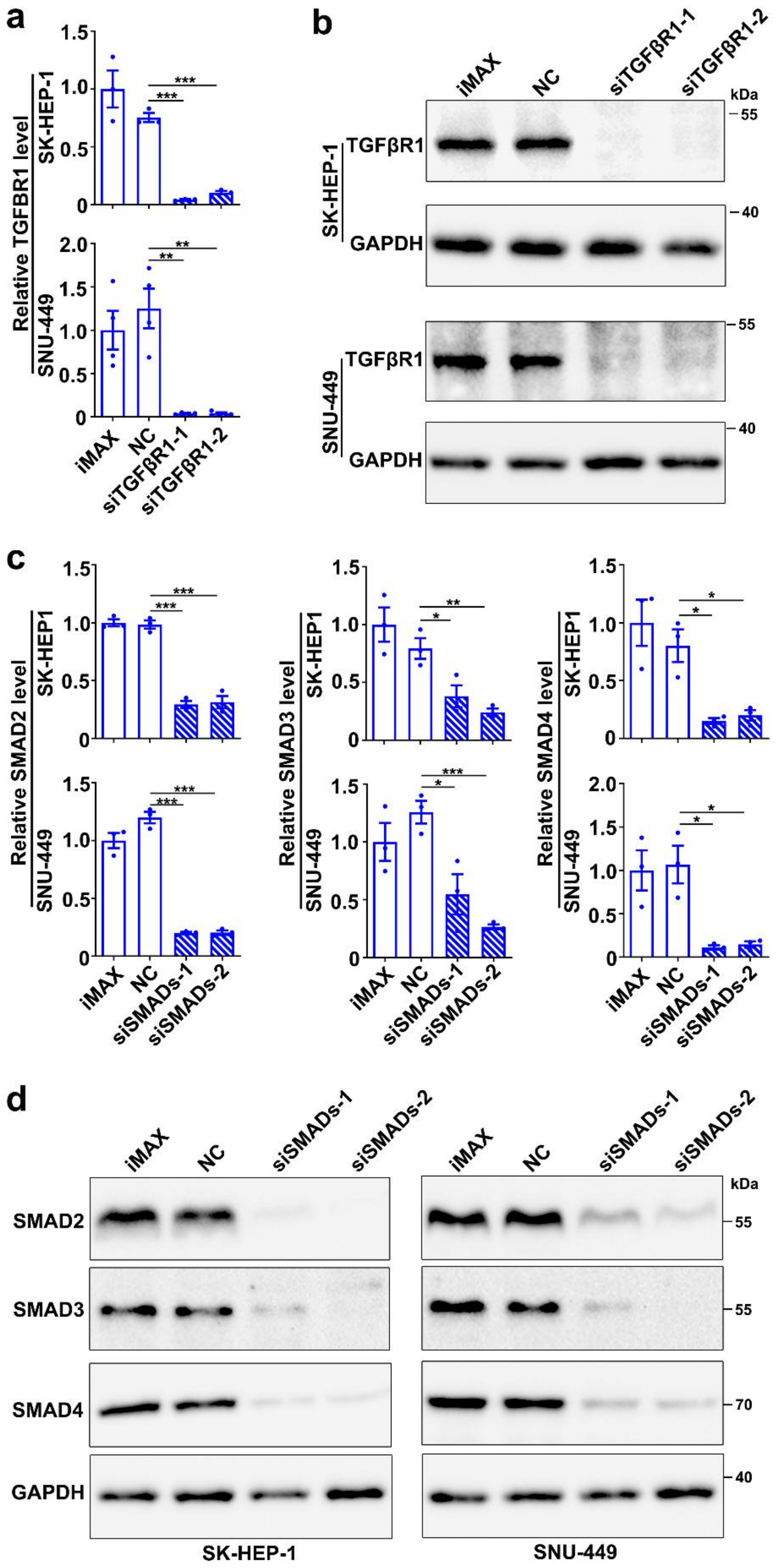

Supplementary Figure S2. Silencing of key components of the TGFβ signaling pathway by siRNAs. (a, b) The knockdown effect of siRNAs targeting TGFβR1. **(c, d)** The

knockdown effect of siRNAs targeting SMAD2, SMAD3 or SMAD4. For (a-d), cells were transfected with the indicated RNA duplexes for 24 hours, and then incubated with TGF-β for 24 hours, followed by qPCR (a, c) or western blotting (b, d). iMAX, cells exposed to Lipofectamine RNAiMAX without RNA duplexes. NC, cells transfected with the negative control RNA duplex. siTGFβR1-1 and siTGFβR1-2, cells transfected with siRNA targeting different sequences of TGFβR1. siSMADs-1 and siSMADs-2, cells transfected with the mixture of siRNAs targeting SMAD2, SMAD3 or SMAD4. U6 and GAPDH were used as internal controls for qPCR and western blotting, respectively. Error bar: SEM from at least three independent experiments. *, $P < 0.05$; **, $P < 0.01$; ***, $P < 0.001$.

Supplementary Fig. S3

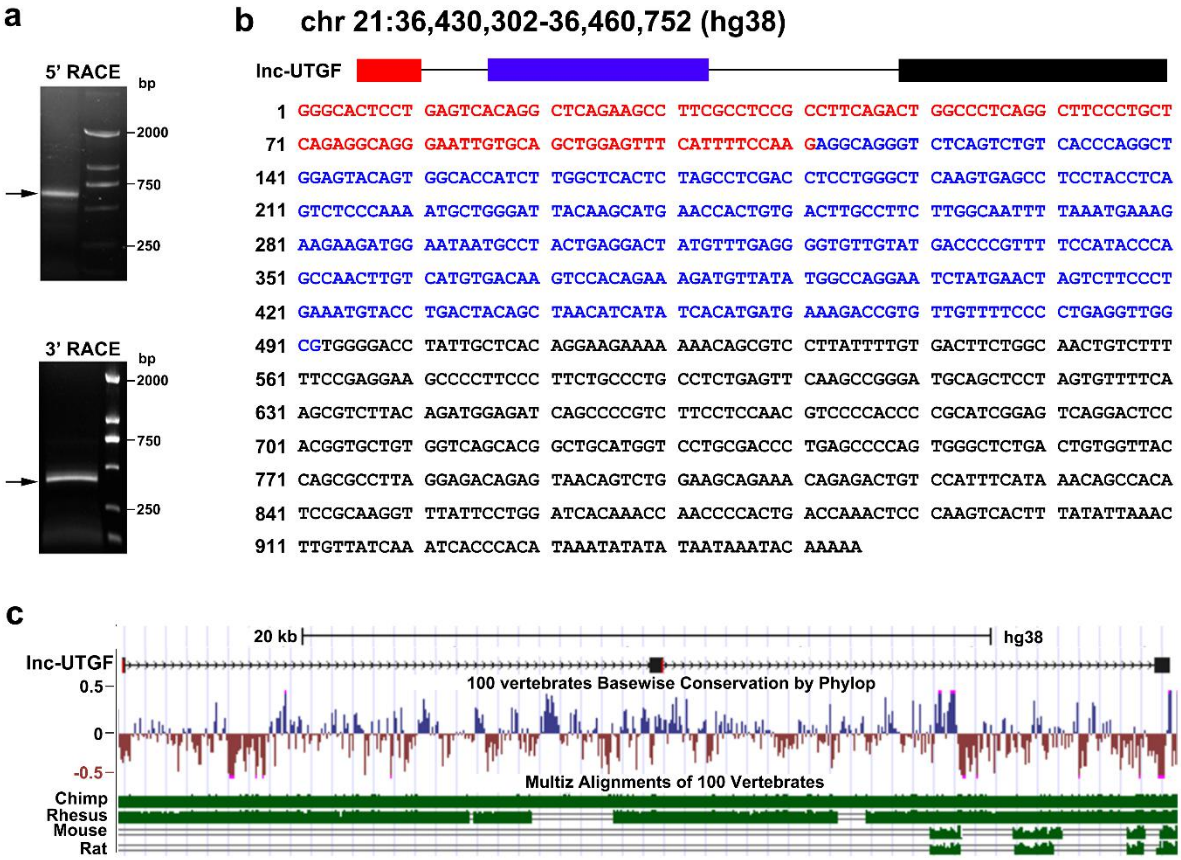

Supplementary Figure S3. Identification of the full-length transcript of lnc-UTGF. **(a)** The PCR products of 5'RACE (*upper panel*) and 3'RACE (*lower panel*) were indicated by the arrows. **(b)** The structure of human lnc-UTGF gene and the sequence of lnc-UTGF transcript. Three exons are indicated by different colors. **(c)** The sequence conservation of lnc-UTGF. Schematic representation of lnc-UTGF gene loci was obtained from the UCSC Genome Browser (http://genome.ucsc.edu/). Green bars represent the sequence conservation in the indicated species.

Supplementary Fig. S4

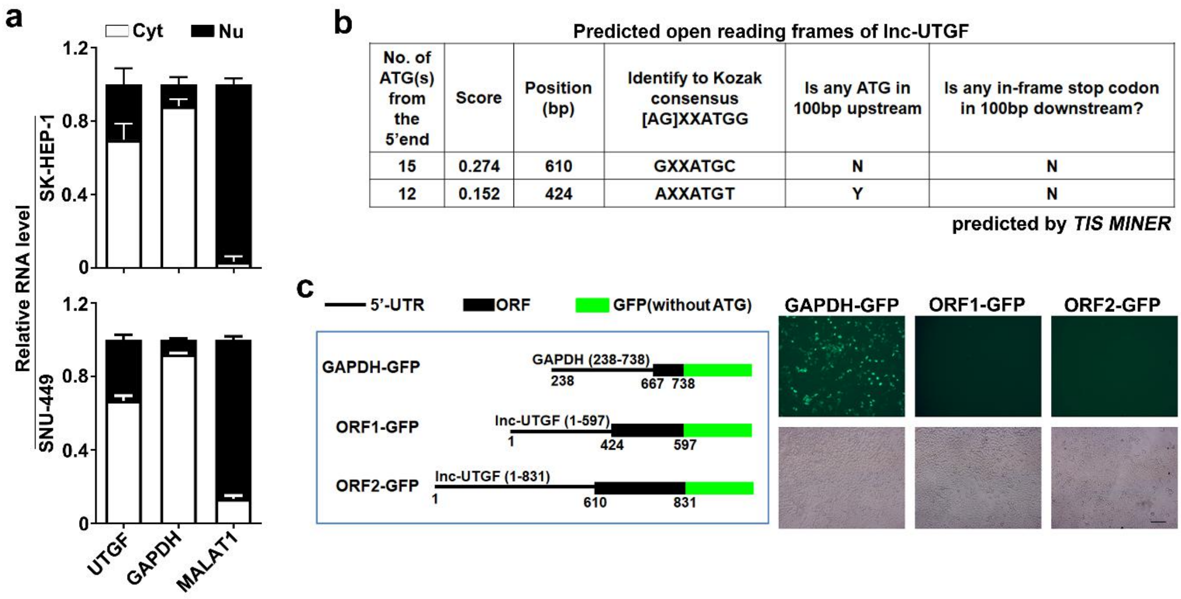

Supplementary Figure S4. Lnc-UTGF is located in the cytoplasm and has no protein-coding capacity. (a) Lnc-UTGF was mainly located in the cytoplasm. The cytoplasm and nucleus of cells were fractioned and then subjected to qPCR. GAPDH, mainly located in the cytoplasm; MALAT1, mainly located in the nucleus. **(b, c)** The predicted ORFs in the lnc-UTGF gene did not show coding ability. The putative open reading frames (ORF) in lnc-UTGF were predicted by the TIS MINER (b, http://dnafsminer.bic.nus.edu.sg/Tis.html). Score < 0.6 means no coding ability. For the *left panel* in (c), a schematic diagram of the expression vectors is shown. The predicted ORF of lnc-UTGF, together with its 5'UTR was fused in-frame to the N-terminus of the GFP-coding sequence (without ATG). The GAPDH-GFP fusion protein was used as a positive control. For the *right panel* in (c), 293T cells were transfected with the indicated plasmids for 48 hours, then analyzed under bright-field and fluorescence microscopy. Cells showed green indicated expression of GFP fusion protein. Scale bar, 100 μm. Error bar: SEM from at least three independent

experiments.

Supplementary Fig. S5

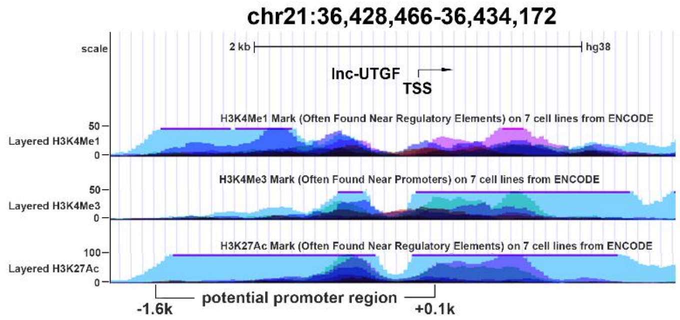

Supplementary Figure S5. The features of the predicted lnc-UTGF promoter. ChIP-seq profiles of H3K4Me1, H3K4Me3 and H3K27Ac are visualized using the UCSC genome browser (http://genome.ucsc.edu/). The transcription direction and the potential promoter region of lnc-UTGF are indicated. TSS, transcription start site.

Supplementary Fig. S6

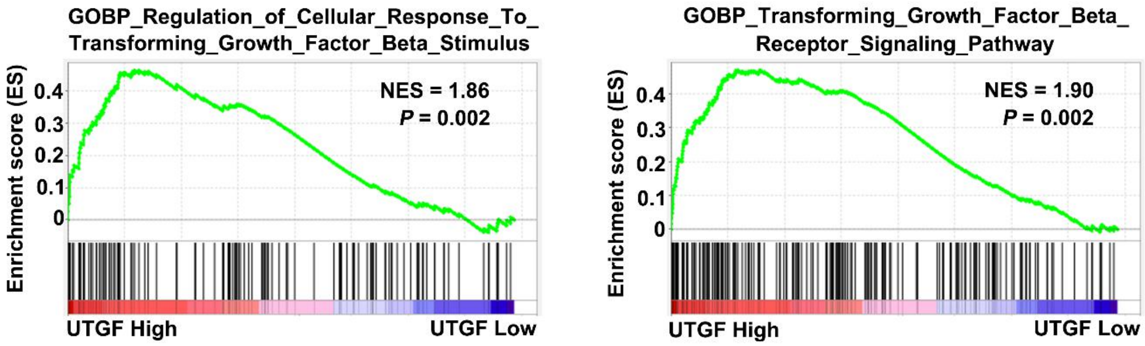

Supplementary Figure S6. Genes regulating the response to TGF-β and genes in the TGF-β receptor pathway are significantly enriched in the group with high lnc-UTGF level. Gene Set Enrichment Analysis (GSEA) was performed using the transcriptome data of human HCC tissues derived from The Cancer Genome Atlas (TCGA). From a total of 199 human HCC tissues, data from 50 cases with the highest lnc-UTGF level (UTGF High) and 51 cases with the lowest lnc-UTGF level (UTGF Low) were used for GSEA.

Supplementary Fig. S7

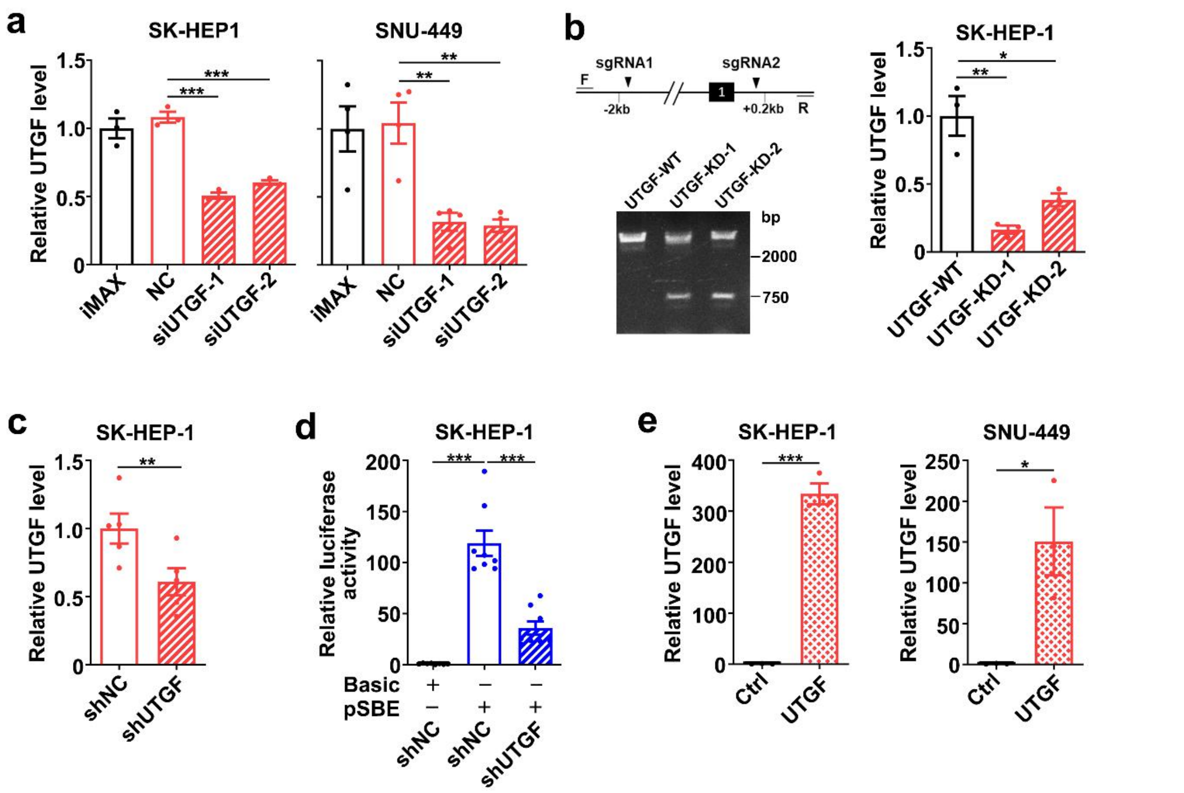

Supplementary Figure S7. Knockdown or overexpression of lnc-UTGF in hepatoma cells. **(a)** Knockdown of cellular lnc-UTGF by siRNA. Cells were transfected with the indicated RNA duplexes for 48 hours, followed by qPCR. siUTGF-1 and siUTGF-2, cells transfected with siRNA targeting different sequences of lnc-UTGF. **(b)** Heterozygous knockout of endogenous lnc-UTGF by CRISPR-cas9 system. UTGF-WT, monoclonal cell line with wild-type promoter of lnc-UTGF; UTGF-KD-1 and -2, two monoclonal cell lines with heterozygous knockout of lnc-UTGF promoter. The arrowheads and the grey lines indicate the position of sgRNAs and PCR primers, respectively. The black rectangle represents the first exon of lnc-UTGF transcript. **(c)** Knockdown of endogenous lnc-UTGF by shRNA. Cells stably expressing shUTGF (shUTGF) and its control cells (shNC) were subjected to qPCR. **(d)** Knockdown of lnc-UTGF attenuated the effect of TGF-β in stimulating pSBE reporter activity. SK-shUTGF and SK-shNC sublines were transfected with the indicated vectors for 36 hours,

then treated with TGF-β for another 12 hours, followed by luciferase activity analysis. Basic, pGL3-basic vector. pSBE, a luciferase reporter bearing twelve tandem SBEs. **(e)** Ectopic expression of lnc-UTGF in hepatoma cells. Cells stably expressing lnc-UTGF or its control cells (Ctrl) were subjected to qPCR. U6 was used as an internal control. Error bar: SEM from at least three independent experiments. *, $P < 0.05$; **, $P < 0.01$; ***, $P < 0.001$.

Supplementary Fig. S8

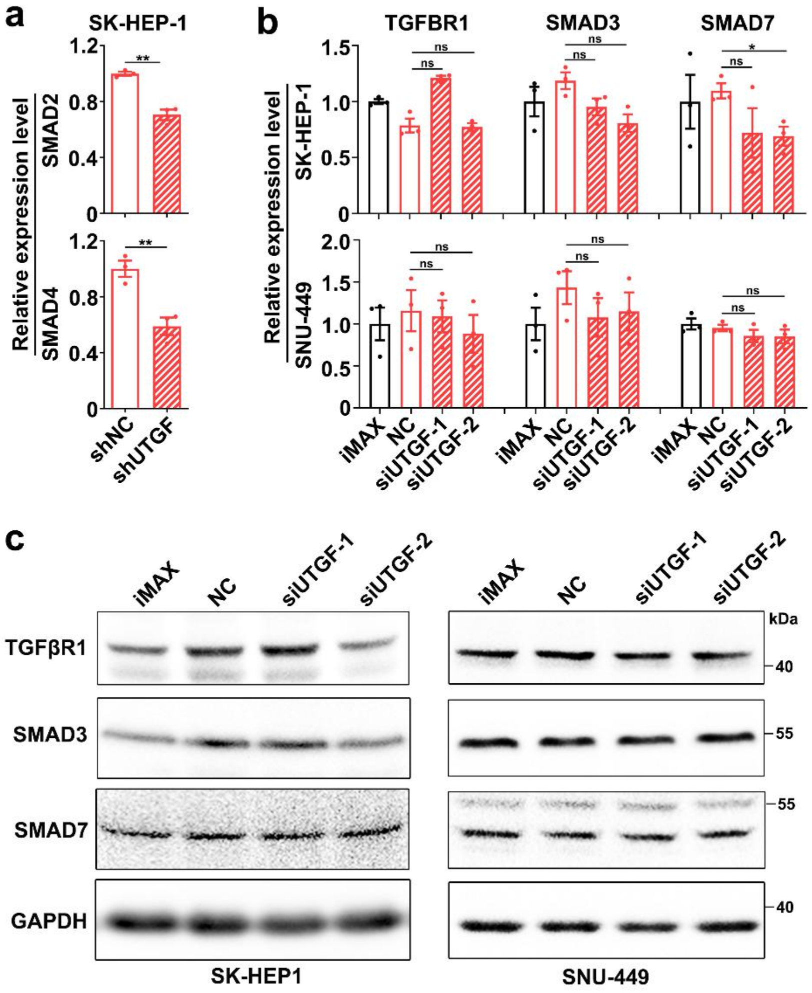

Supplementary Figure S8. The effect of lnc-UTGF silencing on the key components of the TGF-β/SMAD signaling. (a) Silencing lnc-UTGF decreased the mRNA levels of SMAD2 and SMAD4. SK-shUTGF and SK-shNC sublines were subjected to qPCR. **(b, c)** Silencing lnc-UTGF did not affect the mRNA and protein levels of TGFβR1, SMAD3 and SMAD7. Cells that were transfected with the indicated RNA duplexes for 48 hours were subjected to qPCR (b) or western blotting (c). U6 and GAPDH were used as internal controls for qPCR and western blotting, respectively. Error bar: SEM from at least three independent experiments. *, $P < 0.05$; **, $P < 0.01$; ns, not significant.

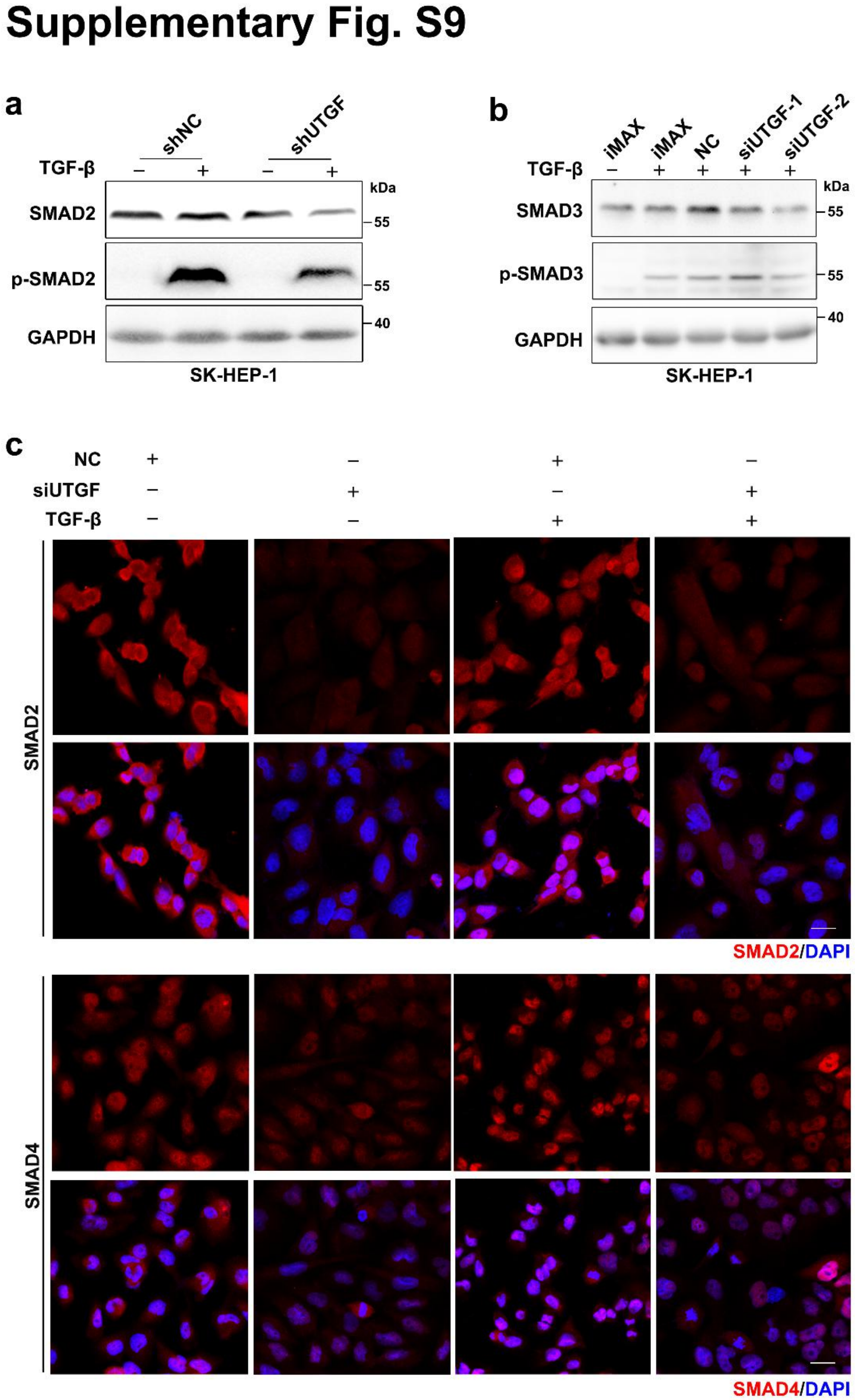

Supplementary Figure S9. Silencing lnc-UTGF decreases the levels of total and phosphorylated SMAD2. (a) Silencing lnc-UTGF decreased the levels of total and phosphorylated SMAD2 protein. **(b)** Silencing lnc-UTGF did not affect the levels of total or

phosphorylated SMAD3 protein. Cells with stable knockdown of lnc-UTGF and their control cells (a), or cells transfected with the indicated RNA duplexes for 48 hours (b) were untreated or treated with TGF-β for 1 hour, followed by western blotting. GAPDH was used as internal controls for western blotting. **(c)** Both nuclear and cytoplasmic SMAD2 and SMAD4 proteins were decreased in siUTGF-transfectants. SK-HEP-1 cells transfected with the indicated RNA duplexes for 48 hours were untreated or treated with TGF-β for 1 hour, followed by immunofluorescence staining. + or −, cells with (+) or without (−) the indicated treatment. Scale bar, 25 μm.

Supplementary Fig. S10

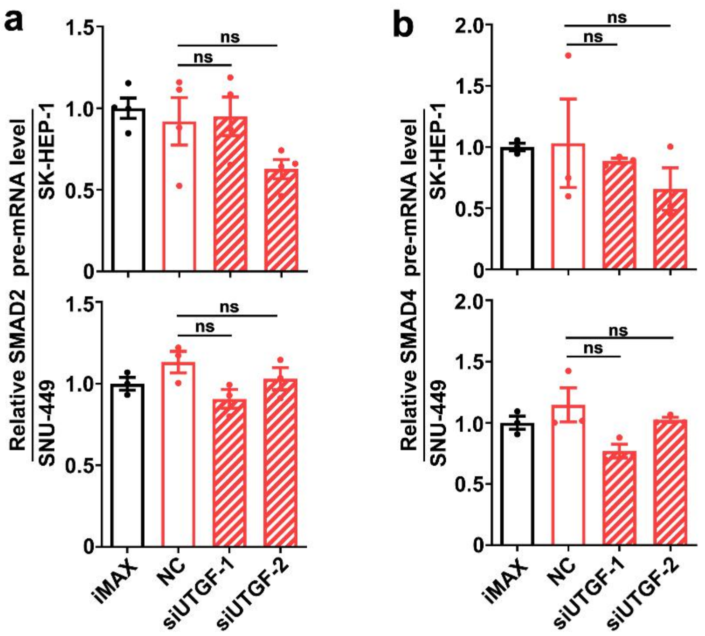

Supplementary Figure S10. Silencing lnc-UTGF has no impact on the precursor mRNA levels of SMAD2 and SMAD4. Cells that were transfected with the indicated RNA for 48 hours were subjected to qPCR for the precursor mRNA levels of SMAD2 (a) and SMAD4 (b). U6 was used as an internal control. Error bar: SEM from at least three independent experiments. ns, not significant.

Supplementary Fig. S11

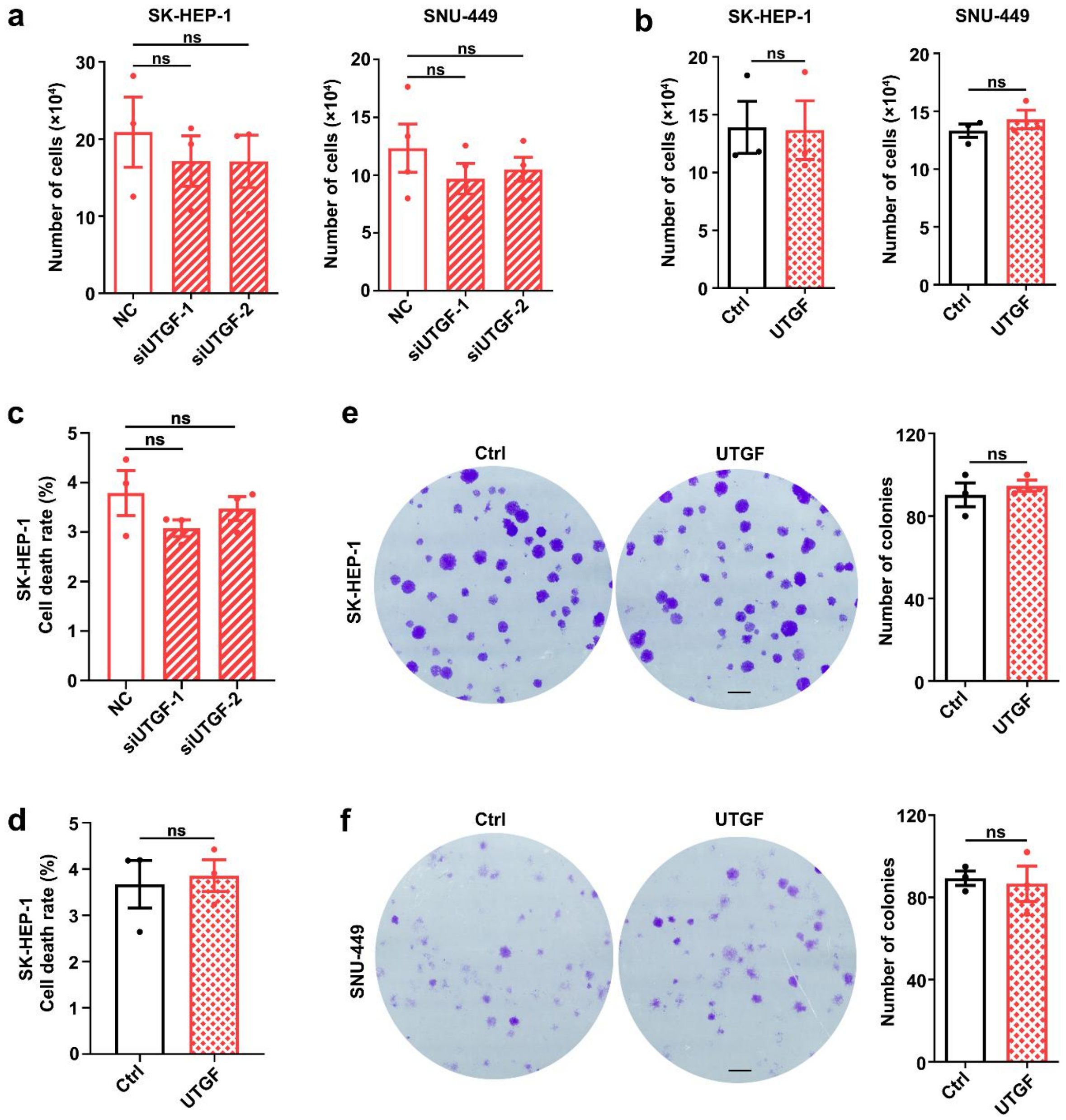

Supplementary Figure S11. Lnc-UTGF does not affect cell proliferation and apoptosis. (a, b) Lnc-UTGF did not affect cell growth. Cells transfected with the indicated RNA (a) or infected with the indicated lentivirus (b) were seeded in a 24-well plate for 72 hours, and the number of cells was counted. **(c, d)** Lnc-UTGF did not affect cell apoptosis. Cells transfected with the indicated RNA (c) or infected with the indicated lentivirus (d) for 48 hours were subjected to DAPI staining. **(e, f)** Lnc-UTGF had no impact on colony formation. The viable

cells infected with the indicated lentivirus were placed in a 6-well plate and maintained in complete medium for 10 days. Colonies were fixed in methanol and stained with crystal violet. The number of the colonies was counted. Error bar: SEM from at least three independent experiments. ns, not significant. Scale bar, 2.5 mm.

Supplementary Fig. S12

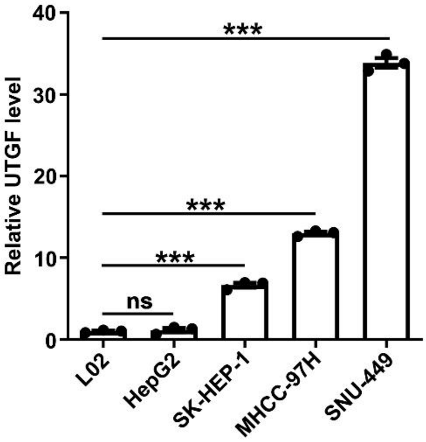

Supplementary Figure S12. Lnc-UTGF expression in different cell lines. Lnc-UTGF levels in L02, HepG2, SK-HEP-1, MHCC-97H and SNU-449 cells were detected by qPCR. Error bar: SEM from at least three independent experiments. ***, $P < 0.001$. ns, not significant.

Supplementary Fig. S13

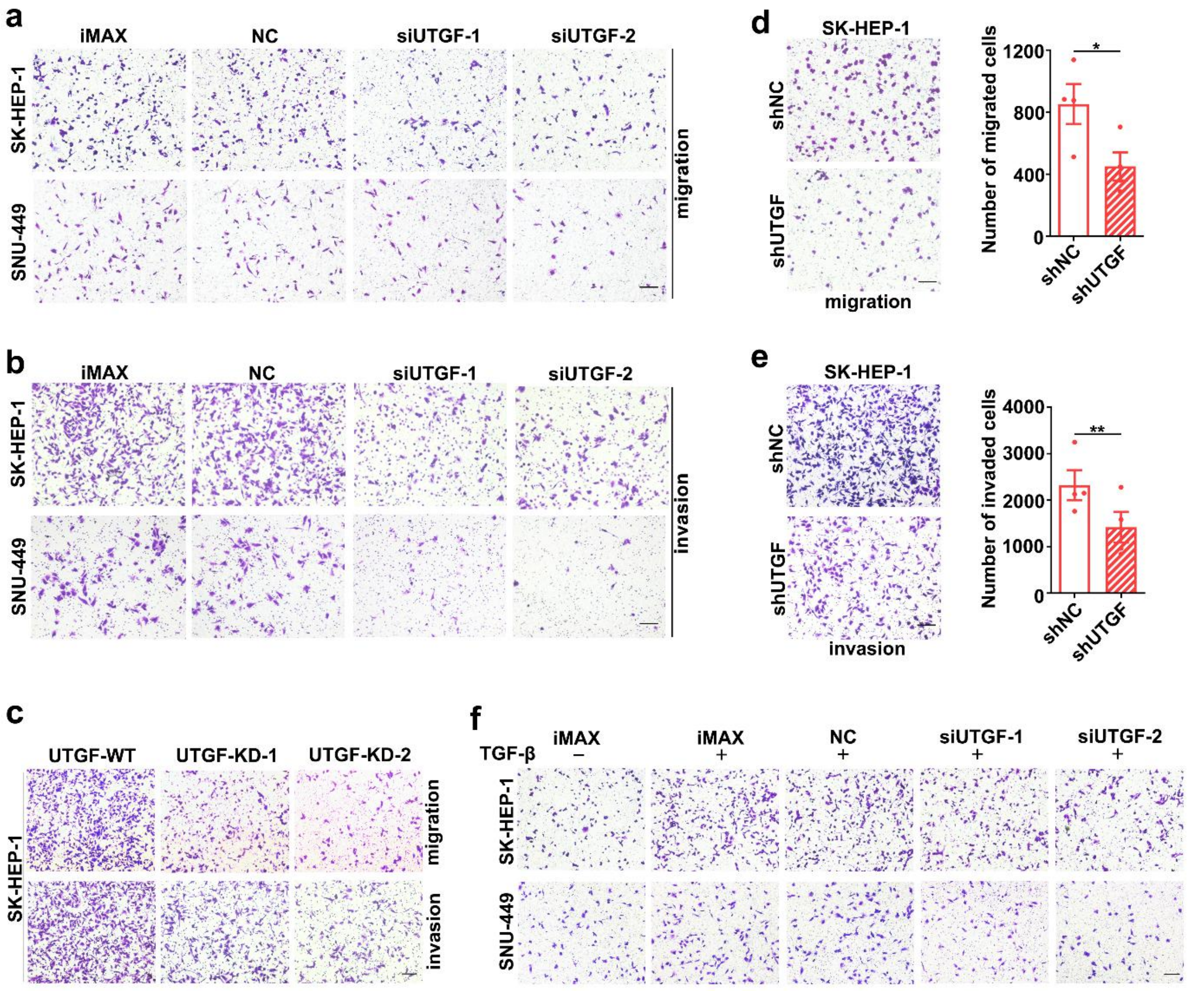

Supplementary Figure S13. Effects of lnc-UTGF silencing in the migration and invasion of hepatoma cells. (a-e) Silencing lnc-UTGF suppressed migration and invasion of hepatoma cells. Cells transfected with the indicated RNA duplexes for 36 hours (a-b), sublines with heterozygous knockout of lnc-UTGF (UTGF-KD-1 and -2) and their control line (UTGF-WT; c) or subline with stable knockdown of lnc-UTGF (shUTGF) and its control line (shNC; d-e) were examined. **(f)** Silencing lnc-UTGF attenuated the TGF-β-stimulated migration of hepatoma cells. Cells that were transfected with the indicated RNA duplexes for 24 hours were incubated without or with TGF-β for another 24 hours. For (**a-f**), cells were added to transwell chambers without (for migration) or with (for invasion) matrigel coatings and incubated for 10 hours, followed by staining with crystal violet. All the migrated/invaded

cells were counted. + or −, cells with (+) or without (−) the indicated treatment. Error bar: SEM from at least three independent experiments. *, $P < 0.05$; **, $P < 0.01$. Scale bar, 100 μm.

Supplementary Fig. S14

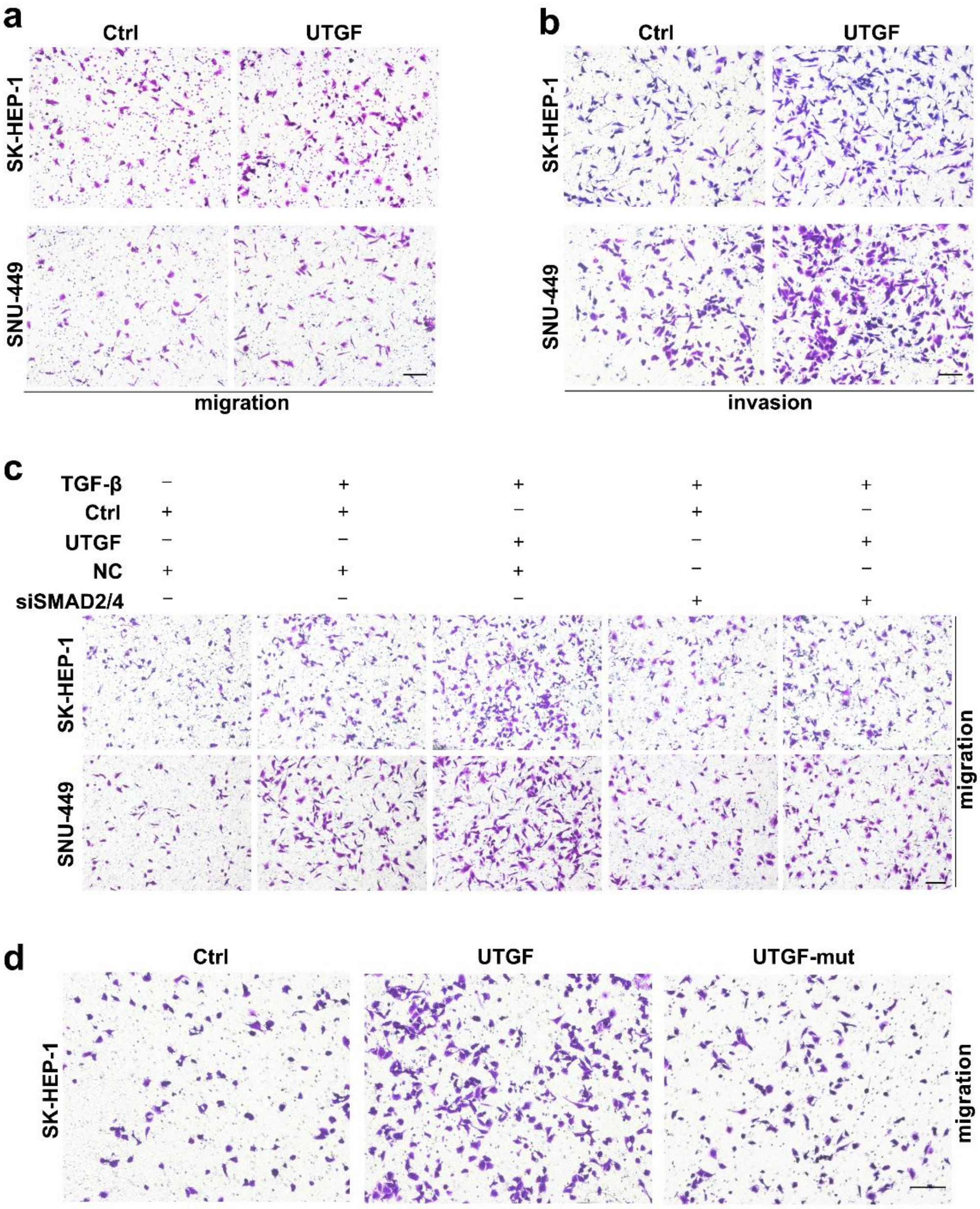

Supplementary Figure S14. Effects of lnc-UTGF overexpression in migration and invasion of hepatoma cells. (a, b) Ectopic expression of lnc-UTGF promoted migration and invasion of hepatoma cells. Cells stably expressing lnc-UTGF and its control cells (Ctrl) were examined. **(c, d)** Silencing SMAD2/4 or mutation of the SMAD2/4-binding sites in lnc-UTGF attenuated the effect of lnc-UTGF in promoting migration of hepatoma cells. For (c), cells stably expressing lnc-UTGF and its control cells (Ctrl) were transfected with the indicated

RNA duplexes for 24 hours, then incubated without or with TGF-β for 24 hours. For (d), cells stably expressing lnc-UTGF with wild-type or mutant SMAD2/4-binding sites (lnc-UTGF-mut) were incubated with TGF-β for 24 hours. + or −, cells with (+) or without (−) the indicated treatment. For (a-d), cells were added to transwell chambers without (for migration) or with (for invasion) matrigel coatings and incubated for 10 hours, followed by staining with crystal violet. Scale bar, 100 μm.

Supplementary Fig. S15

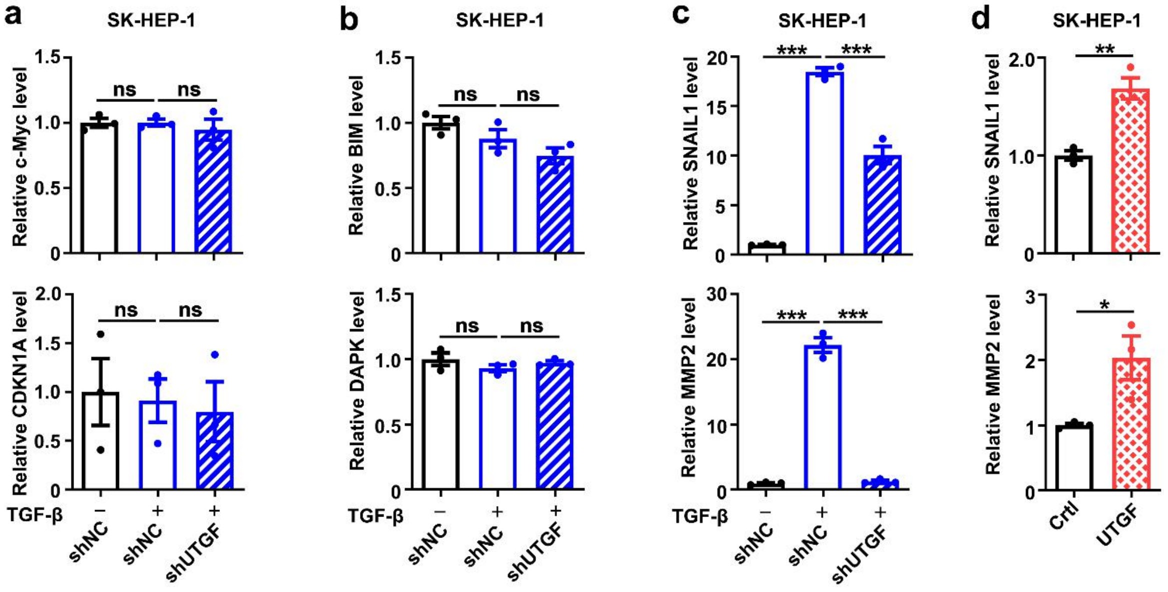

Supplementary Figure S15. The effect of lnc-UTGF on the downstream genes of the TGF-β/SMAD signaling. (a, b) TGF-β treatment or silencing lnc-UTGF did not change the levels of the TGF-β downstream molecules that were involved in cell proliferation and apoptosis. **(c)** Silencing lnc-UTGF attenuated the effect of TGF-β in increasing SNAIL1 and MMP2 levels. For (a-c), SK-shNC and SK-shUTGF sublines were incubated without or with TGF-β for 36 hours, followed by qPCR. **(d)** Overexpressing lnc-UTGF increased the mRNA levels of SNAIL1 and MMP2. Cells stably expressing lnc-UTGF and the control cells (Ctrl) were subjected to qPCR. + or −, cells with (+) or without (−) the indicated treatment. U6 was used as an internal control. Error bar: SEM from at least three independent experiments. *, $P < 0.05$; **, $P < 0.01$; ***, $P < 0.001$; ns, not significant.

Supplementary Fig. S16

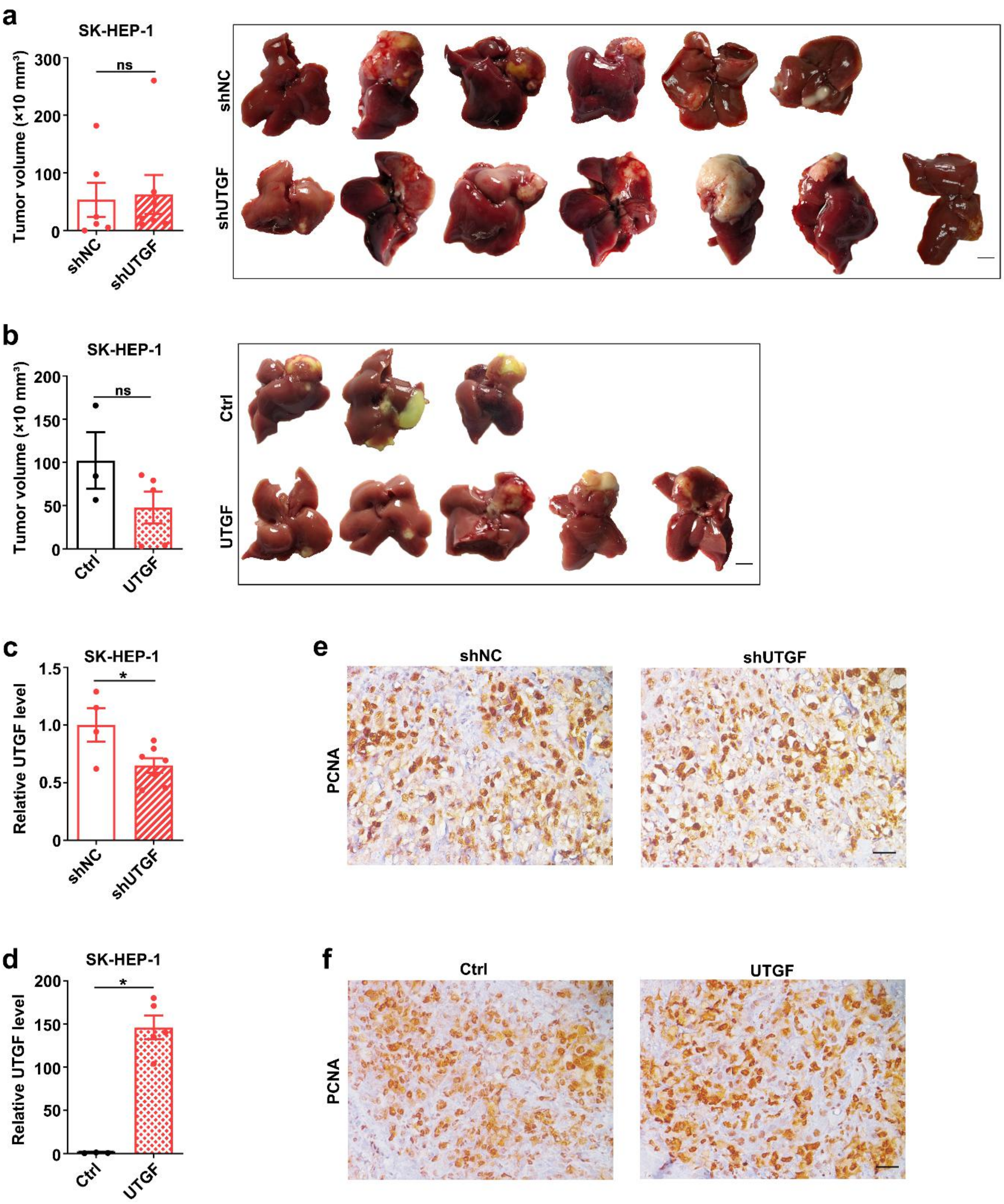

Supplementary Figure S16. Lnc-UTGF does not affect the growth of tumor xenografts in BALB/c nude mice. (a, b) Neither silencing nor overexpressing lnc-UTGF affected the growth of tumor xenografts. SK-shNC (n = 6) and SK-shUTGF (n = 7) (a), or SK-Ctrl (n = 3) and SK-UTGF (n = 5) (b) sublines were inoculated under the capsule of the left hepatic lobe

of BALB/c nude mice. Scale bar, 5 mm. **(c, d)** The expression of lnc-UTGF in tumor xenografts. The lnc-UTGF levels in tumor xenografts derived from lnc-UTGF-silencing (c) or lnc-UTGF-overexpressing (d) cells were detected by qPCR. U6 was used as an internal control. **(e, f)** Neither silencing nor overexpressing lnc-UTGF affected the proliferation of tumor cells. PCNA was stained to label the proliferated tumor cells. Scale bar, 25 μm. Error bar, SEM. *, $P < 0.05$. ns, not significant.

Supplementary Fig. S17

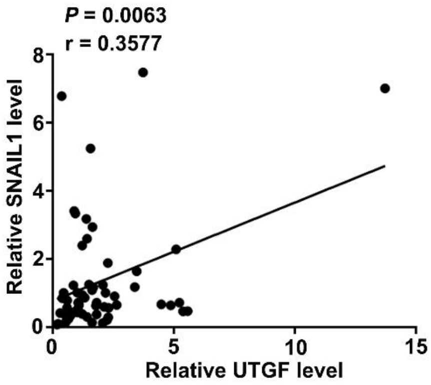

Supplementary Figure S17. The lnc-UTGF level is positively correlated with the mRNA level of SNAIL1. The levels of lnc-UTGF and SNAIL1 were examined in 57 HCC tissues by qPCR. Pearson's correlation coefficient analysis was employed.

Supplementary Fig. S18

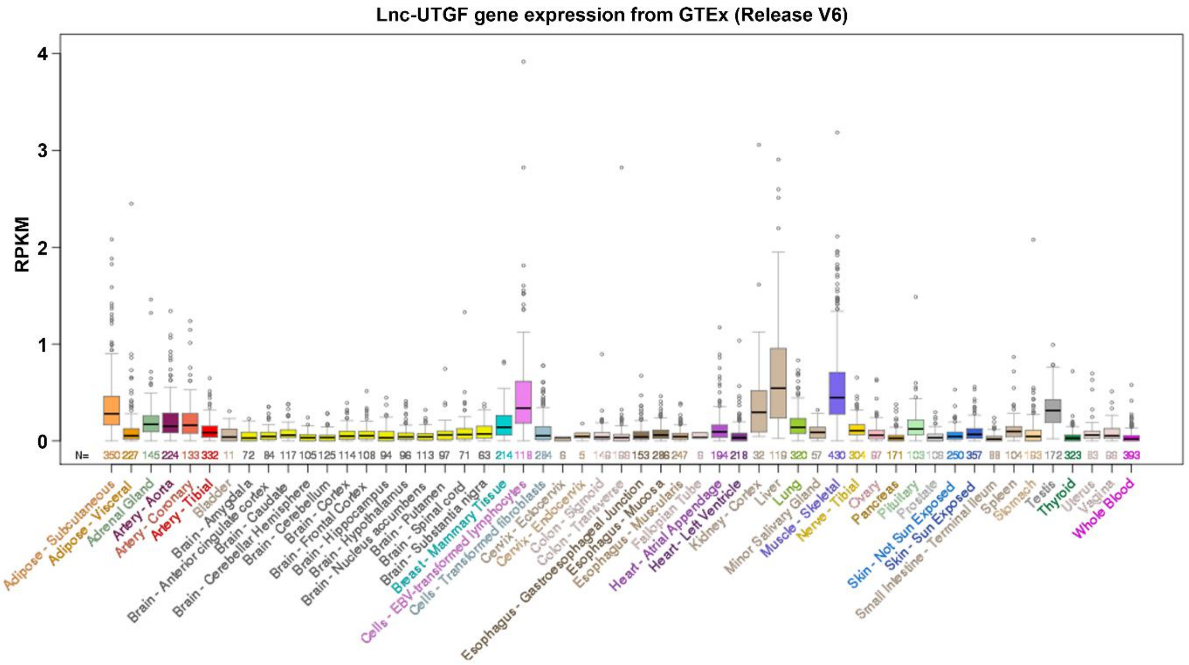

Supplementary Figure S18. Lnc-UTGF is universally expressed in different tissues. The lnc-UTGF levels in different tissues were analyzed. The transcriptome data derived from the Genotype-Tissue Expression (GTEx) database were extracted from http://genome.ucsc.edu/ website.

Supplementary Table S1. Association between lnc-UTGF Expression and Clinical Features[a]

Characteristics		UTGF-high group[b] (N=74)		UTGF-low group[b] (N=88)		P^c
		No.	%	No.	%	
Age-year	>50	20	37.7	33	62.3	0.106
	≤50	54	49.5	55	50.5	
**Gender**	Male	69	49.3	71	50.7	**0.016**
	Female	5	22.7	17	77.3	
HBV infection	Yes	72	45.9	85	54.1	0.582
	No	2	40	3	60	
Tumor size	≥5cm	56	50.5	61	49.5	0.235
	＜5cm	18	40	27	60	
Tumor number	Multiple	20	51.3	19	48.7	0.267
	Single	54	43.9	69	56.1	
Tumor capsule	No	46	48.9	48	51.1	0.178
	Yes	27	40.3	40	59.7	
	NA	1				
Cirrhosis	Yes	38	43.7	49	56.3	0.382
	No	35	47.3	39	52.7	
	NA	1				
Alpha-fetoprotein	≥400ng/ml	42	49.4	43	50.6	0.128
	<400ng/ml	29	39.2	45	60.8	
	NA	3				
**Portal vein tumor thrombus**	Yes	18	62.1	11	37.9	**0.040**
	No	56	42.1	77	57.9	
**Edmondson grade**	I/II	27	35.5	49	64.5	**0.009**
	III/IV	47	55.3	38	44.7	
	NA			1		
**TNM**	II/III[d]	42	54.5	35	45.5	**0.023**
	I	32	37.6	53	62.4	

[a] Analysis was conducted on 162 cases. The lnc-UTGF level was examined by qPCR and normalized to U6 level.

[b] The 54th percentile of the lnc-UTGF level in 162 HCC tissues was chosen as the cut-off value for separating the lnc-UTGF-low level group (n = 88) from the lnc-UTGF-high level group (n = 74).

[c] The association between lnc-UTGF level and clinical features of HCC patients was analyzed by chi-square test.

[d] 23 stage II and 54 stage III cases were included.

Supplementary Table S2. Sequences of RNA and DNA Oligonucleotides

Name	Sense Strand/Sense Primer (5'-3')	Antisense Strand/Antisense Primer (5'-3')
**siRNA Duplexes**		
siUTGF-1	CCACAUCCGCAAGGUUUAU dTdT	AUAAACCUUGCGGAUGUGGdCdT
siUTGF-2	GCAAGGUUUAUUCCUGGAU dTdT	AUCCAGGAAUAAACCUUGC dGdG
siSMAD2-1	GAAUUGAGCCACAGAGUAAdTdT	UUACUCUGUGGCUCAAUUCdCdT
siSMAD2-2	GCCCUCACUCACUGUAGAU dTdT	AUCUACAGUGAGUGAGGGC dTdG
siSMAD3-1	CGUCAACACCAAGUGCAUCdTdT	GAUGCACUUGGUGUUGACGUU
siSMAD3-2	CCGCAUGAGCUUCGUCAAA dTdT	UUUGACGAAGCUCAUGCGG dAdT
siSMAD4-1	GCCAGCUACUUACCAUCAU dTdT	AUGAUGGUAAGUAGCUGGC dTdG
siSMAD4-2	GCCUCCCAUUUCCAAUCAU dTdT	AUGAUUGGAAAUGGGAGGC dTdG
siTGFβR1-1	GGAGAUUGUUGGUACCCAAdTdT	UUGGGUACCAACAAUCUCCdAdT
siTGFβR1-2	CCAUUGAUAUUGCUCCAAA dTdT	UUUGGAGCAAUAUCAAUGG dTdA
NC	UUGUACUACACAAAAGUACUG	GUACUUUUGUGUAGUACAGUU
**Primers for RACE**		
GSP1	GATTACGCCAAGCTTATGCGGGGTGGGGACGTTGGAG	
GSP2	GATTACGCCAAGCTTCTCAGGGGAAAACAACGCGGTCT	
GSP3	TGGGGACCTATTGCTCACA	
GSP4	TTCCCTTCTGCCCTGCCTCT	
**Primers for Gene cloning (Restriction enzyme sites were underlined)**		
lnc-UTGF (pCDH)	CGGAATTCGGGCACTCCTGAGTCACAGGC	CGGGATCCTTTTTGTATTTATTATATATATTTATGTGGG
lnc-UTGF (pCDH-S1m)	CGGAATTCGGGCACTCCTGAGTCACAGGC	CGATTTAAATTTTTTGTATTTATTATATATATTTATGTGGG
lnc-UTGF (pc3-sense)	CGGGATCCGGGCACTCCTGAGTCACAGGC	CGGAATTCTTTTTGTATTTATTATATATATTTATGTGGG
lnc-UTGF (pc3-antisense)	CGGAATTCGGGCACTCCTGAGTCACAGGC	CGGGATCCTTTTTGTATTTATTATATATATTTATGTGGG

Supplementary Table S2. Sequences of RNA and DNA Oligonucleotides (continued)

Name	Sense Strand/Sense Primer (5'-3')	Antisense Strand/Antisense Primer (5'-3')
**Oligos for CRISPR/cas9**		
sgRNA1	CTCAAGTGACCCACCCGCCT	
sgRNA2	TGTAACACAAGCCTGCGGCG	
proUTGF	TTTCTGGGCTTGACTG	GGTACTGGTGGTTAGGACTTCA
**Oligos for shUTGF**		
shUTGF-oligo1	AATTCGCAAGGTTTATTCCTGGATTTCAAGAGA	ATCCAGGAATAAACCTTGCG
shUTGF-oligo2	ATCCAGGAATAAACCTTGCTTTTTG	GATCCAAAAAGCAAGGTTTATTCCTGGATTCTCTTGAA
**Primers for lnc-UTGF promoter plasmids**		
-1.6k-F	CGAGCTCTTACGCGTGCTAGCAGGCTGACTTGAGGGATGAC	
-1.2k-F	CGAGCTCTTACGCGTGCTAGCGGACTGTGTTATTGGAGAGGC	
-0.8k-F	CGAGCTCTTACGCGTGCTAGCTTCCCAGTCAGCCCTAGG	
-0.4k-F	CGAGCTCTTACGCGTGCTAGCGATCATGCCGCTCTTGTTTTG	
-0.1k-F	CGAGCTCTTACGCGTGCTAGCAAGAAACACAATCTGGCCGC	
+0.1k-R	CAGTACCGGAATGCCAAGCTTAACTCCAGCTGCACAATTCC	
mutSBE	GCCTGCCTGGCCTCAGCTCTGGCCTCTG	CAGAGGCCAGAGCTGAGGCCAGGCAGGC
delSBE	GAGTGGCTACACCGGCAGCTCTGGCCTCTG	CAGAGGCCAGAGCTGCCGGTGTAGCCACTC
**Primers for ChIP**		
lnc-UTGF	ACCTCTAAGTCGCCAAACCA	GCGGCCAGATTGTGTTTCTT
GAPDH	TACTAGCGGTTTTACGGGCGCACGT	TCGAACAGGAGGAGCAGAGAGCGAA
**Primers for RT-PCR**		
lnc-UTGF	CTCAGAGGCAGGGAATTGTG	TGGGAGACTGAGGTAGGAGG
TGFβR1	TTGGCAAAGGTCGATTTGGA	AGAGCTGAGTCCAAGTACCA

Supplementary Table S2. Sequences of RNA and DNA Oligonucleotides (continued)

Name	Sense Strand/Sense Primer (5'-3')	Antisense Strand/Antisense Primer (5'-3')
**Primers for RT-PCR**		
SMAD2	CGTCTCCAGGTATCCCATCG	GTCATCCAGAGGCGGAAGTT
SMAD3	TATCCCCGAATCCGATGTCC	GTGGAATGTCTCCCCGACG
SMAD4	CAGATAGCATCAGGGCCTCA	CAGAAGGGTCCACGTATCCA
SMAD7	TGTGCAAAGTGTTCAGGTGG	CGGGTATCTGGAGTAAGGAGG
c-MYC	TCTTCCCCTACCCTCTCAAC	TCCAGACTCTGACCTTTTGC
CDKN1A	GTGTCGGTGGGGCTCATC	GCTTGGCGTTATCGTGGAC
BIM	ATTACCAAGCAGCCGAAGAC	TCCGCAAAGAACCTGTCAAT
DAPK	AGAAATTCAAGAAGTTTGCAG	GTCTTCCTCATCCAGAGTAT
SNAIL1	TGCGTCTGCGGAACCTG	GGACTCTTGGTGCTTGTGGA
MMP2	AGGGCACATCCTATGACAGC	ATTTGTTGCCCAGGAAAGTG
U6	CTCGCTTCGGCAGCACA	AACGCTTCACGAATTTGCGT
